# DNA sequence characterisation and phylogeography of *Lymnaea cousini *and related species, vectors of fascioliasis in northern Andean countries, with description of *L. meridensis *n. sp. (Gastropoda: Lymnaeidae)

**DOI:** 10.1186/1756-3305-4-132

**Published:** 2011-07-12

**Authors:** M Dolores Bargues, Patricio Artigas, Messaoud Khoubbane, Santiago Mas-Coma

**Affiliations:** 1 Departamento de Parasitología, Facultad de Farmacia, Universidad de Valencia, Av. Vicente Andrés Estellés s/n, 46100 Burjassot - Valencia, Spain

## Abstract

**Background:**

Livestock fascioliasis is a problem throughout Ecuador, Colombia and Venezuela, mainly in Andean areas where the disease also appears to affect humans. Transmission patterns and epidemiological scenarios of liver fluke infection have shown to differ according to the lymnaeid vector snail species involved. These Andean countries present the vectors *Lymnaea cousini*, *L. bogotensis *and *L. ubaquensis*, unknown in the rest of Latin America. An exhaustive combined haplotype study of these species is performed by means of DNA sequencing of the nuclear ribosomal 18S RNA gene, ITS-2 and ITS-1, and mitochondrial DNA *cox*1 gene.

**Results:**

The conserved 5.8S rDNA sequence corroborated that no pseudogenes are involved in the numerous non-microsatellite/minisatellite-related indels appearing between the ITS-2 and ITS-1 sequences when comparing different *L. cousini *- *L. bogotensis *populations. Sequence analyses and phylogenetic reconstruction methods including other lymnaeid vector species show that (i) *L. bogotensis *is a synonym of *L. cousini*, (ii) *L. ubaquensis *is a synonym of *Pseudosuccinea columella*, and (iii) populations of *L. cousini *hitherto known from Venezuelan highlands indeed belong to a new species for which the name *L. meridensis *n. sp. is proposed. This new species is described and a complete phenotypic differentiation provided.

**Conclusions:**

ITS-2, ITS-1 and *cox*1 prove to be good markers for specimen classification and haplotype characterisation of these morphologically similar lymnaeids in endemic areas. Analysis of the 18S gene and phylogenetic reconstructions indicate that *L. cousini *and *L. meridensis *n. sp. cluster in an evolutionary line different from the one of *P. columella*, despite their external resemblance. This suggests an evolutionary phenotypic convergence related to similar environments and which has given rise to frequent specimen misclassification. Body size and phylogenetic relationships of *L. meridensis *n. sp. with well-known vectors as *Lymnaea cousini *and *P. columella*, as well as with *Galba*/*Fossaria *species, suggest that the new species may participate in disease transmission to both animals and humans in altitude areas during the yearly window in which temperatures are higher than the *F. hepatica *minimum development threshold. The involvement of *L. cousini *and *P. columella *in the transmission and geographical/altitudinal distribution of fascioliasis in these Andean countries is analysed.

## Background

The two liver fluke species *Fasciola hepatica *and *F. gigantica *(Trematoda: Fasciolidae) cause fascioliasis, a highly pathogenic disease which appears to emerge in many countries of Latin America, Europe, Africa and Asia at present [1]. This parasitic disease has been recognised as a very important veterinary problem in livestock since long ago [2]. Its public health impact at the human level has, however, only been ascertained from the 1990s [3-5].

In the two host life cycle of fasciolids, livestock species play an important reservoir role. Transmission studies have shown that different species, such as sheep, cattle, pig and donkey, represent similar infection sources when considering the infectivity of the metacercarial infective stage from their respective origins [6,7]. On the contrary, the specificity of fasciolid species regarding freshwater snail species of the family Lymnaeidae (Gastropoda) [8], represents a crucial factor in establishing not only the geographical distribution of the disease in both animals and humans, but also prevalences and intensities due to more or less appropriate ecological characteristics (population dynamics, anthropophilic characteristics, type of water bodies, etc.) of the different lymnaeid intermediate host or vector species. That is why different lymnaeid species appear linked to the different transmission patterns and epidemiological scenarios of this very heterogeneous disease in humans [1,4]. Additionally, both lymnaeid snails and the larval stages of fasciolids have been shown to be highly dependent on climatic and environmental characteristics [9-11]. This explains the relationships of fascioliasis with climate change effects recently observed in different areas [12,13].

In the Americas, fascioliasis is only caused by *F. hepatica*, due to the absence of lymnaeids of the genus *Radix *which act as transmitters of *F. gigantica *[8]. In South America, human fascioliasis endemic areas appear mainly related to high altitude areas of Andean countries, where the transmission of *F. hepatica *has been shown to be increased [14]. Two different patterns are included [4]: (i) the Altiplano pattern with transmission throughout the year due to lymnaeid vectors linked to permanent water bodies [15] as in the human hyperendemic areas of Bolivia [15,16] and Peru [17], and (ii) the valley pattern with seasonal transmission due to lymnaeid vectors more linked to temporary water bodies, including situations as those in Chile [18] and Peru [19-21].

Human and animal fascioliasis is also known to be a great problem in northern Andean countries such as Ecuador [22-24], Colombia [25-32] and Venezuela [33,34]. In all these areas, epidemiological and transmission characteristics should *a priori *fit the valley pattern. Among the lymnaeid species acting as vectors of fascioliasis in these countries, *Lymnaea cousini *Jousseaume, 1887 is one of the species mainly involved in the transmission in high altitude areas in Ecuador [24,35], Colombia [25,36] and Venezuela [37,38]. Shell morphology, ecological characteristics and seasonal population dynamics of this species [25,39-41] explain why it may be confused, when dealing with young specimens, with other lymnaeids, mainly vector species of the so-called *Galba*/*Fossaria *group [42]. These facts are also related to the different lymnaeid species having been proposed to be synonyms of *L. cousini*: *L. raphaelis *Jousseaume, 1887 described from Azuay, South of Cuenca, Ecuador; *L. selli *Preston, 1907 and *L. bogotensis *Pilsbry, 1935, both originally described from Bogota, Colombia; and *L. ubaquensis *Piaget, 1914 only known from Laguna Ubaque, Cundinamarca, Colombia [37,39,43,44].

The problems in specimen classification and species distinction in the aforementioned vector groups is a good example of the wide confusion in which the family Lymnaeidae is immersed, due to the large intraspecific variability and the insufficiency of efficient classification characteristics in shell and morphoanatomy on which to rely [8]. Fortunately, in recent years, genetic studies by means of DNA marker sequences have proved to be very useful in assessing not only the classification of lymneid snail specimens, but also the validity of the species and its phylogenetic relationships with other taxa of the family in the way for a natural systematic and taxonomic classification of the family. Additionally, these molecular tools have shown to be appropriate tools to analyse the fasciolid species/lymnaeid species specificity, by providing deep distinction capacity of populations and geographic strains by combined haplotyping when using different markers of the nuclear ribosomal DNA (rDNA) and mitochondrial DNA (mtDNA) together [45]. Therefore, a worldwide initiative to clarify vector species within Lymnaeidae by molecular tools was launched in parallel to multidisciplinary studies to assess human fascioliasis in the different continents [1]. Markers used so far and having shown their usefulness at different levels are: (i) within the rDNA operon, the 18S gene [42,46,47], ITS-2 [8,14,42,48-50] and ITS-1 [14,48,51]; (ii) within the mtDNA genome, the 16S [50,52,53], and *cox*1 [42,54].

The purpose of the present study is to characterise the vector species *L. cousini*, *L. bogotensis *and *L. ubaquensis *from Ecuador, Colombia and Venezuela by means of DNA sequences of 18S, ITS-2, ITS-1 and *cox*1, compare their different populations, reconstruct their phylogenetic relationships with other close vector species of the family Lymnaeidae in the Americas, and analyse its geographical distribution and transmission role regarding human and animal fascioliasis. The results of the molecular analyses, made by using specimens of the type localities of each Latin American lymnaeid species in order to be systematically conclusive, indicate that a new species is present in Venezuela. For this species the name *Lymnaea meridensis *n. sp. is proposed and a diagnostic description is provided.

## Methods

### Lymnaeid snail materials

**Figure 1:**
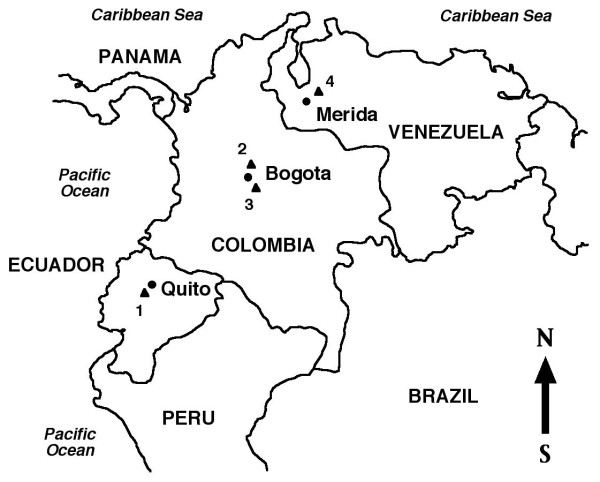
**Geographical distribution of lymnaeid sampling localities**. Map of northern Andean countries showing localities where lymnaeids were collected: 1 = *Lymnaea cousini *from Chanchu-Yacu, Chillogallo, Quito, Ecuador; 2 = *L. bogotensis *from Zipaquira, savannah of Bogota, Cundinamarca, Colombia; 3 = *L. ubaquensis *from Laguna de Ubaque, Cundinamarca, Colombia; 4 = *L. meridensis *n. sp. from Laguna Mucubaji, Merida State, Venezuela.

The snail specimens studied were from the following lymnaeid species and geographical origins (Figure 1):

1.- *Lymnaea cousini *Jousseaume, 1887 from the type locality of Chanchu-Yacu, Chillogallo, Quito, Ecuador [55] (0°18'54.9'' S, 78°34'2.3'' W - 3,036 m a.s.l.) and from Laguna Mucubaji, Merida State, Venezuela (8°47'51.8'' N, 70°49'32.4'' W - 3,550 m a.s.l.).

2.- *L. bogotensis *Pilsbry, 1935 from the type locality of the savannah of Bogota, Cundinamarca, Colombia [56] (snails collected in the locality of Zipaquira; 4°59'46.4'' N, 74°0'6.6'' W - 2,567 m a.s.l.); *L. selli *Preston, 1907 indeed corresponds to the same species and identical type locality [39].

3.- *L. ubaquensis *Piaget, 1914 from the type locality of Laguna de Ubaque, Cundinamarca, Colombia [57] (4°29'11.3'' N, 73°56'11.0'' W - 1,884 m a.s.l.).

4.- *Pseudosuccinea columella *(Say, 1817) from Rio Piedras, Puerto Rico (18°23'45.5'' N, 66°3'24.5'' W - 9 m a.s.l.), used for comparison purposes; a Caribbean origin was selected due to the core distribution of this species in that area [58].

DNA was extracted from more than one specimen of a given population when this was deemed necessary for sequence verification. Only snails that appeared free of helminth infection were used in the molecular analyses. To reduce further the risk of contamination of DNA from helminths (which are more likely to be localized in other tissues), DNA was only isolated from the foot of each snail.

### Molecular techniques

#### DNA extraction

Snail feet fixed in 70% ethanol were used for DNA extraction procedures. After dissection under a microscope, half of the foot was suspended in 400 μl of lysis buffer (10 mM Tris-HCl, pH 8.0, 100 mM EDTA, 100 mM NaCl, 1% sodium dodecyl sulfate SDS) containing 500 μg/ml Proteinase K (Promega, Madison, WI, USA) and digested for 2 hr at 55°C with alternate shaking each 15 min. The procedure steps were performed according to methods outlined previously [8,42,46]. Total DNA was isolated according to the phenol-chloroform extraction and ethanol precipitation method [59]. The pellet was dried and resuspended in 30 μl sterile TE buffer (pH 8.0). This suspension was stored at -20°C until use.

#### DNA sequence amplification

DNA sequences were amplified by PCR using 4-6 μl of genomic DNA for each 50 μl PCR reaction, according to methods outlined previously [8,14,42,46]. Each one of the five DNA markers were PCR amplified independently for each lymnaeid specimen and each PCR product was sequenced for a bona-fide haplotype characterisation. A set of 8 conserved oligonucleotide primers was used for the amplification of five superimposed fragments of the 18S ribosomal RNA gene using specific primers and a standard protocol [42,47] to amplify specific 18S rDNA regions. The rDNA spacers ITS-2 and ITS-1 were amplified using primers designed in conserved positions of 5.8S and 28S rRNA genes and 18S and 5.8S rRNA genes of several eukaryote Metazoa species, respectively [8,42,51]. A mitochondrial DNA *cox*1 gene fragment was amplified using universal primers [60]. Amplifications were generated in a Mastercycle ep*gradient *(Eppendorf, Hamburg, Germany), by 30 cycles of 30 sec at 94°C, 30 sec at 50°C and 1 min at 72°C, preceded by 30 sec at 94°C and followed by 7 min at 72°C for ITS-2 and ITS-1, and by 40 cycles of 30 sec at 90°C, 1 min at 48°C and 1 min at 72°C, preceded by 2.5 min at 94°C and followed by 10 min at 72°C for *cox*1. Ten μl of each PCR product was checked by staining with ethidium bromide on 1% Nusieve^® ^GTG agarose (FMC) gel electrophoresis, using the Molecular Weight Marker VI (Boehringer Mannheim) at 0.1 μg DNA/μl as control.

#### Purification and quantification of PCR products

Primers and nucleotides were removed from PCR products by purification on Wizard™ PCR Preps DNA Purification System (Promega, Madison, WI, USA) according to the manufacturer's protocol and resuspended in 50 μl of 10 mM TE buffer (pH 7.6). The final DNA concentration was determined by measuring the absorbance at 260 and 280 nm.

#### DNA sequencing

The sequencing of the entire 18S rRNA gene, the complete rDNA ITS-2 and ITS-1 and, and the fragment of the mtDNA *cox*1 gene was performed on both strands by the dideoxy chain-termination method [61]. It was carried out with the Taq dye-terminator chemistry kit for ABI 373A and ABI 3700 capillary system (Perkin Elmer, Foster City, CA, USA), using PCR primers.

#### Pseudogene absence verification

To assure that no pseudogenes were involved in the sequences of the intergenic region of the different lymnaeid populations analysed, the 5.8S rRNA gene was amplified independently using primers designed in conserved regions of the 18S and ITS-2 rDNA of several lymnaeid species [8,42,51,62]. To further assure accurateness of the sequences obtained in the way to discriminate between lymnaeid populations, additional amplifications were carried out using population-specific primers designed in the sequences of the ITS-2 region previously obtained. Amplifications were generated in a Mastercycle ep*gradient *(Eppendorf, Hamburg, Germany), by 35 cycles of 30 sec at 94°C, 40 sec at 55°C and 2 min at 72°C, preceded by 3 min at 94°C and followed by 3 min at 72°C for the 5.8S rDNA region.

#### DNA haplotype nomenclature

The codes for the sequences obtained follow the standard nomenclature proposed for lymnaeid snails previously [1,45,51]. It shall be noted that haplotype codes are only definitive in the case of complete sequences. When dealing with fragments or incomplete sequences, haplotype codes are provisional.

### Software programs

#### Sequence alignments

Sequences were aligned using CLUSTAL-W version 1.8 [63] and MEGA 4.0 [64], and assembly was made with the Staden Package [65]. Subsequently, minor corrections were manually introduced for a better fit of nucleotide correspondences in microsatellite sequence regions. Homologies were performed using the BLASTN programme from the National Center for Biotechnology information web site http://www.ncbi.nlm.nih.gov/BLAST. Genetic distances were measured using parameters provided by PAUP v.4.0b10 [66].

#### Sequence comparisons

The following sequences from GenBank-EMBL have been used for comparison analyses:

- 18S rRNA gene: complete sequences of *Lymnaea (Lymnaea) stagnalis *[EMBL: Z73984], *Lymnaea (Stagnicola) palustris *[EMBL: Z73983], *Omphiscola glabra *[EMBL: Z73982], *Galba truncatula *[EMBL: Z73985] [46]; *L. cubensis *[EMBL: Z83831] [42,47]; *L. viatrix *and *L. neotropica *both species with the same sequence [EMBL: AM412222] [42]; *Pseudosuccinea columella *[GenBank: EU241866] [67]; *Radix auricularia *[EMBL: Z73980] and *R. balthica *[EMBL: Z73981] [46]. Other incomplete sequences available in the GenBank have not been used to avoid problems in comparative sequence analyses.

- rDNA ITS-2: *L*. (*S*.) *palustris palustris *[EMBL: AJ319620], *L*. (*S*.) *palustris turricula *[EMBL: AJ319618], *L*. (*S*.) *fuscus *[EMBL: AJ319621] and *Catascopia occulta *[EMBL: AJ319642] [8,49] (*C. occulta *has been recently proposed to be a younger synonym of *C. terebra *[68] although molecular confirmation is still pending); *L. cubensis *H1 from Cuba [EMBL: AM412223] and H2 from USA [EMBL: FN182200], *L. viatrix *[EMBL: AM412224] and *L. neotropica *[EMBL: AM412225], including respective type localities for each one [42]; *G. truncatula *H1 [EMBL: AJ296271], H2 [EMBL: AJ243017] and H3 (= *L. viatrix sensu *Ueno *et al*., 1975; = *L. cubensis sensu *Ueno *et al*., 1975) [EMBL: AJ272051] [8,14,42]; *P. columella *from Cuba [GenBank: AY186751] [69].

- rDNA ITS-1: *L*. (*S*.) *palustris palustris *[EMBL: AJ626849], *L*. (*S*.) *palustris turricula *[EMBL: AJ626853], *L*. (*S*.) *fuscus *[EMBL: AJ626856] and *C. occulta *[EMBL: AJ626858] [51]; *L. cubensis *HA from Cuba [EMBL: AM412226] and HB from USA [EMBL: FN182202], *L. viatrix *[EMBL: AM412227], and *L. neotropica *[EMBL: AM412228], including respective type localities for each one [42]; *G. truncatula *HA [EMBL: AJ243018], HB [EMBL: AJ296270] and HC (= *L. viatrix sensu *Ueno *et al*., 1975; = *L. cubensis sensu *Ueno *et al*., 1975) [EMBL: AJ272052] [8,42,51]; *P. columella *from Cuba [GenBank: AY186751] [69].

- mtDNA *cox*1 gene: *L. cubensis cox*1-a [EMBL: AM494009], *L. viatrix cox*1-a [EMBL: AM494010], *L. neotropica cox*1-a [EMBL: AM494008], all three from respective type localities [42]; *L. neotropica cox*1-b from Argentina [EMBL: FN356741] [62]; *G. truncatula cox*1-a from Spain [EMBL: AM494011] [42]; *G. truncatula *from Germany [GenBank: EU818799] [70]; and *P. columella *from Australia [GenBenk: AY227366] [54].

#### Representation of the 18S rRNA secondary structure

The previously published secondary structure prediction for *Limicolaria kambeul *18S rRNA [71] based on the general eukaryote 18S rRNA secondary structure [72] was used and extended to encompass lymnaeid sequences.

### Phylogenetic inference

Phylogenies were inferred from DNA sequences using maximum-likelihood (ML) estimates with PAUP. ML parameters such as model, base frequencies, transition/transversion ratio (ts/tv), the shape parameter for the gamma distribution, and the proportion of invariant sites, were optimised using the hierarchical likelihood ratio test (hLRT) and the Akaike information criterion (AIC) [73,74], implemented in Modeltest 3.7 [75]. Starting branch lengths were obtained using the least-squares method with ML distances.

To provide an assessment of the reliability of the nodes in the ML tree, three methods were used. First, a bootstrap analysis using 1000 replicates was made with fast-heuristic search in PAUP. Second, a distance-based phylogeny using the neighbor-joining (NJ) algorithm [76] with the ML pairwise distances was obtained and statistical support for the nodes was evaluated with 1000 bootstrap replicates, with and without removal of gapped positions. Third, a Bayesian phylogeny reconstruction procedure was applied to obtain posterior probabilities (BPP) for the nodes in the ML tree, by using the same evolutionary model as above, implemented in MrBayes 3.1 [77] with four chains during 1,000,000 generations and trees were sampled every 100 generations. The first 1000 trees sampled were discarded ("burn-in") and clade posterior probabilities (PP) were computed from the remaining trees. Alternative methods of phylogenetic reconstruction allowing an evaluation of the support for each node were also applied. A distance-based phylogeny using the NJ algorithm with LogDet distances was obtained. Statistical support for the nodes was evaluated with 1000 bootstrap replicates.

Due to the several limitations recently shown by mtDNA coding genes for interspecific sequence analyses in invertebrates [78-80], phylogenetic reconstruction was only made from combined sequences of ITS-2 and ITS-1 as the markers considered to be best for the analysis of relationships between species belonging to different genera, as has already been verified in Lymnaeidae [45].

Phylogenetic analyses were performed after adding reference sequences of ITS-2 and ITS-1 of lymnaeid rDNA stored in the GenBank database (see species and Acc. Nos. used in list noted above in chapter of sequence comparisons). The intergenic region sequence (Genbank: AY030361) [81] including both ITSs of a planorbid species, *Biomphalaria pfeifferi*, was used as outgroup.

### Phenotypic study

Shells of lymnaeids were measured, according to traditional malacological methods [82,83], using a computerized image-analysis system [84]. This system was based on a DXC-930P colour video camera (Sony, Tokyo) fitted to a stereomicroscope, and connected to a computer running image analysis software (ImageProH Plus 4.5; Media Cybernetics Inc., Silver Spring, MD).

For anatomical studies, adult lymnaeids were collected in the field and allowed to relax overnight in water containing menthol. They were then immersed for 40 s in hot water (70°C) before transfer to water at room temperature. The soft parts were drawn from the shells with forceps applied to the cephalopedal mass, and fixed in slightly modified Railliet-Henry's fluid (930 ml distilled water, 6 g NaCl, 50 ml 40% formalin, and 20 ml glacial acetic acid). The fixed snails were then dissected under a stereomicroscope, so that drawings of the reproductive system could be made using a camera lucida [85].

## Results

### 18S rRNA Gene

**Figure 2:**
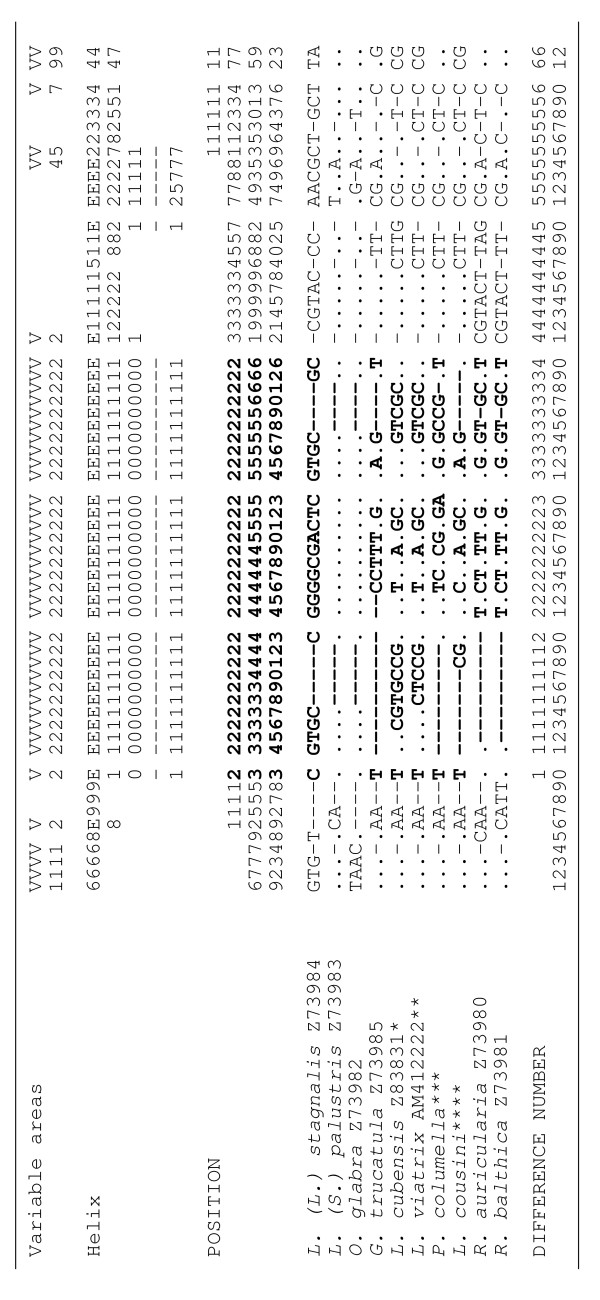
**Nucleotide differences in a total of 62 variable positions found in the complete 18S rDNA sequence of the lymnaeid species compared and their location in the secondary structure.** Helix, Position and Difference number = numbers to be read in vertical. Position = numbers refer to positions obtained in the alignment made with MEGA 4.0. Identical = .; Indel = -. Bold text corresponds to helix E10-1 of the variable area V2 where differences in the 18S rRNA gene of Lymnaeidae are concentrated [46]. Accession Nos. = [EMBL: Z73980-Z73985] [46]; [EMBL: Z83831] [47]; [EMBL: AM4122222] [42]; *L. cousini *and *P. columella *from present study. Sequence correspondences: * *L. cubensis*, *L. viatrix *and *L. cousini *without definitive genus ascription; ** 18S identical in *L. viatrix *and *L. neotropica *[42]; *** 18S identical in *P. columella *and *L. ubaquensis *(present paper); **** 18S identical in *L. cousini *from Ecuador, *L. bogotensis *and *L. meridensis *n. sp. (= *L. cousini *from Laguna Mucubaji, Venezuela) (present study)

The 18S rDNA sequence of *L. cousini *from Chanchu-Yacu (Ecuador) and Laguna Mucubaji (Venezuela) and the one from *L. bogotensis *from Bogota savannah (Colombia) are identical base to base, with a length of 1848 bp, GC content of 51.70%, and base frequencies of: A = 0.239, G = 0.282, C = 0.235, and T = 0.244. This 18S sequence has been deposited in GenBank under the Accession No. FN598151. The 18S rDNA sequence of *L. ubaquensis *from Laguna Ubaque (Colombia) showed a sequence different to the aforementioned one, but identical to that of *P. columella *from Puerto Rico, with a 1850 bp length and a 51.70% GC content (0.238 A, 0.282 G, 0.235 C, 0.245 T), and which has been deposited under Acc. No. FN598152. When comparing both 18S sequences, a total of 16 nucleotide differences appear, including 11 mutations and 5 insertions/deletions (indels) (see Figure 2).

When comparing the 18S sequence shared by *L. ubaquensis *and *P. columella *with the only *P. columella *18S from Argentina [EU241866] previously available in GenBank, a total of 22 nucleotide differences unexpectedly appeared (6 ts, 4 tv and 12 indels in a 1856 bp-long pairwise alingment), most differences representing singleton polymorphic sites in conserved areas of the secondary structure of this gene.

The multiple sequence alignment, including the ten different 18S sequences of (a) *L. cousini *and *L. bogotensis*, (b) *P. columella *from Puerto Rico and *L. ubaquensis*, the stagnicoline species (c) *L. (L.) stagnalis*, (d) *L. (S.) palustris *and (e) *O. glabra*, the fossarine species (f) *G. truncatula*, (g) *L. cubensis *and (h) *L. viatrix *(identical sequence as in *L. neotropica)*, and the radicine species (i) *R. auricularia *and (j) *R. balthica*, was 1867 bp long, showing a total of 62 variable nucleotide positions (3.32% divergence) (Figure 2). A total of 31 of these 62 polymorphic sites appears grouped between positions 233 and 266, which corresponds to the helix E10-1 of the variable area V2 of the secondary structure. The other modified positions appear isolated in variable areas V1, V2, V4, V5 and V9 and scattered throughout the rest of the 18S sequence (Figure 2).

### rDNA ITS-2

The populations of *L. cousini *from Chanchu-Yacu (Ecuador) and *L. bogotensis *from Bogota savannah (Colombia) show an identical ITS-2 sequence, which is different from the one showed by *L. cousini *from Laguna Mucubaji (Venezuela). These sequences have been deposited in GenBank with the Acc. Nos. FN598153 and FN598154, respectively. Their length and slightly GC biased average nucleotide composition were 506 bp and 57.70% in Ecuador and Colombia, and 457 bp and 58.85% in Venezuela.

The pairwise comparison of these two ITS-2 sequences shows 64 polymorphic sites, including 12 ts, 3 tv and 49 indels and representing a 12.65% divergence (Table 1). Worth mentioning is that the high number of indels is not due to the presence or absence of microsatellites and differences in corresponding repeats. Only 17 indels appear related to microsatellite repeats, all of them located in the 3' sequence end: GGTC (2 times in Ecuador and Colombia, 1 time in Venezuela), GCAG (2 and 1 times respectively), GT (7 and 3), and CGT) (2 and 1).

**Table 1:** Sequence differences detected in pairwise comparisons of ITS-2 and ITS-1 between *Lymnaea cousini *and the most proximal species *L. bogotensis *and *L. meridensis *n. sp. (= *L. cousini *from Laguna Mucubaji, Venezuela)

	Alignment length	Nucleotide differences		Substitutions				Insertions + deletions	
				Transitions		Transversions			
Compared species	No. of bp	**No**.	%	**No**.	%	**No**.	%	**No**.	%
ITS-2:									
*L. cousini *H1^1 ^vs. *L. meridensis *H1^2^	506	64	12.65	12	2.37	3	0.59	49	9.68

ITS-1:									
*L. cousini *HA vs. *L. cousini *HB^3^	593	24	4.05	11	1.85	5	0.84	8	1.33
*L. cousini *HA vs. *L. meridensis *HA^2^	605	71	11.73	14	2.31	9	1.49	48	7.93
*L. cousini *HB^3 ^vs. *L. meridensis *HA^2^	602	68	11.29	10	1.65	10	1.65	48	7.97

Sequence correspondences: ^1 ^ITS-2 identical in *L. cousini *and *L. bogotensis*; ^2 ^*L. meridensis *n. sp. = *L. cousini *from Laguna Mucubaji (Venezuela); ^3 ^*L. cousini *HB = *L. bogotensis*

The ITS-2 sequence of *L. ubaquensis *from Ubaque (Colombia) differs pronouncedly from that of *L. cousini *from Chanchu-Yacu (Ecuador) and *L. bogotensis *from Bogota savannah (Colombia) and also from the one of *L. cousini *from Laguna Mucubaji (Venezuela). On the contrary, when compared to *P. columella *from Puerto Rico, *L. ubaquensis *only shows one mutation, with C or T in the position 6 of the ITS-2 alignment, respectively. These two sequences have been deposited in the GenBank with the Acc. Nos. FN598155 for the population of Puerto Rico and FN598156 for that of Ubaque. When compared to the ITS-2 of *P. columella *from Cuba available in the GenBank (AY186751), a total of 2 mutations and 9 indels appear.

**Figure 3:**
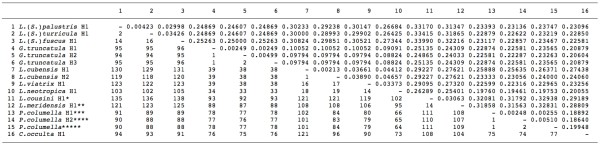
**Pairwise distances between rDNA ITS-2 sequences of the lymnaeid species analysed according to PAUP**. Below diagonal = total character differences; above diagonal = mean character differences (adjusted for missing data). Sequence correspondences: * *L. cousini *H1 = *L. bogotensis*; ** *L. meridensis *n. sp. = *L. cousini *from Laguna Mucubaji (Venezuela); *** *P. columella *H1 corresponds to the population of Puerto Rico; **** *P. columella *H2 = *L. ubaquensis*; ***** *P. columella *[GenBank: AY186751] from Cuba [69]

For an analysis of species relationships, a comparison between these four ITS-2 sequences (*L. cousini *= *L. bogotensis*, *L. cousini *from Venezuela, *P. columella *from Puerto Rico, and *L. ubaquensis *= *P. columella *from Colombia) and those of stagnicolines and fossarines of GenBank was made with a pairwise ITS-2 distance matrix (Figure 3).

### rDNA ITS-1

Each one of the lymnaeid populations studied presented a different ITS-1 sequence. Their length and slightly GC biased average nucleotide composition were: 592 bp and 57.06% in *L. cousini *from Chanchu-Yacu (Ecuador); 587 bp and 56.82% in *L. bogotensis *from Bogota savannah (Colombia); 530 bp long and 58.67%GC in *L. ubaquensis *from Laguna Ubaque (Colombia); and 570 bp and 58.41% in *L. cousini *from Laguna Mucubaji (Venezuela). The four sequences have been deposited under the Acc. Nos. FN598157, FN598158, FN598160 and FN598159, respectively.

The most similar sequences were those of *L. cousini *from Chanchu-Yacu (Ecuador) and *L. bogotensis *from Bogota savannah (Colombia), with only 24 polymorphic sites (11 ts, 5 tv and 8 indels) representing a 4.05% divergence. When the aforementioned two sequences are pairwise compared with *L. cousini *from Laguna Mucubaji (Venezuela), the number of nucleotide differences increases notably (Table 1). None of the numerous indels corresponds to microsatellite repeat differences.

As in the case of ITS-2, the ITS-1 sequence of *L. ubaquensis *is very different from the three aforementioned sequences, but identical to the one of *P. columella *from Puerto Rico. In a pairwise alignment comparison with *P. columella *ITS-1 from Cuba available in the GenBank (AY186751: 527 bp long and 58.44% GC), three indels appear in positions 262, 270 and 276. Worth noting is the presence of A in position 510, in which whether A or G were found in Cuba depending to the susceptibility or resistance characteristics of the population, respectively.

**Figure 4:**
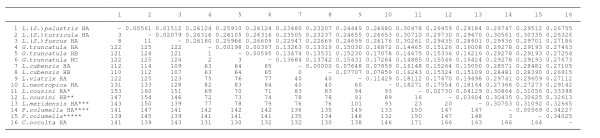
**Pairwise distances between rDNA ITS-1 sequences of the lymnaeid species analysed according to PAUP**. Below diagonal = total character differences; above diagonal = mean character differences (adjusted for missing data). Sequence correspondences: * *L. cousini *HA corresponds to the population of Chanchu-Yacu (Ecuador); ** *L. cousini *HB = *L. bogotensis*; *** *L. meridensis *n. sp. = *L. cousini *from Laguna Mucubaji (Venezuela); **** *P. columella *HA from Puerto Rico = *L. ubaquensis*; ***** *P. columella *[GenBank: AY186751] from Cuba [69]

For an analysis of species relationships, a comparison between these four ITS-1 sequences (*L. cousini*, *L. bogotensis*, *L. cousini *from Venezuela, and *L. ubaquensis *= *P. columella *from Puerto Rico) and those of stagnicolines and fossarines of GenBank was made with a pairwise ITS-1 distance matrix (Figure 4).

#### Putative Intergenic Region Pseudogene Analysis

Only functional 5.8S rDNA sequences were obtained in all individuals and populations analysed of *L. cousini *from Chanchu-Yacu (Ecuador), *L. bogotensis *from Bogota savannah (Colombia), *L. cousini *from Laguna Mucubaji (Venezuela), *L. ubaquensis *from Ubaque (Colombia) and *P. columella *from Puerto Rico. These sequences were all identical in length and nucleotide composition and have been deposited in GenBank with the Acc. Nos. HM560968, HM560969 and HM560970. Their length and slightly GC biased average composition were 154 bp and 55.84%. No methylation-related substitutions (C-T, G-A) were detected. Thus, no degree of polymorphism was observed for the 5.8S rDNA.

#### mtDNA *cox*1

Five different *cox*1 sequences were obtained, all of 672 bp and highly AT-biased. Respective AT contents and GenBank Accession Nos. are: *L. cousini *from Chanchu-Yacu (Ecuador): 69.5% (FN598161); *L. cousini *from Laguna Mucubaji (Venezuela): 69.2% (FN598164); *L. bogotensis *from Bogota savannah (Colombia): two different sequences with 69.6% (FN598162) and 69.8% (FN598163); *L. ubaquensis *from Laguna Ubaque (Colombia) and *P. columella *from Puerto Rico: both share the same sequence, with 69.1% (FN598165).

**Figure 5:**
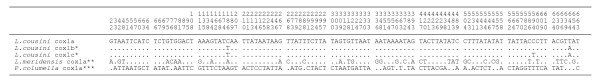
**Nucleotide differences found in the mtDNA *cox*1 sequence of the lymnaeid species studied**. Position = numbers (to be read in vertical) refer to variable positions obtained in the alignment made with MEGA 4.0. Identical = .; Indel = -. Haplotype codes only provisional due to incomplete sequences of the gene. Sequence correspondences: * *L. cousini cox*1b and *cox*1c correspond to two different haplotypes found in *L. bogotensis*; ** *L. meridensis *n. sp. = *L. cousini *from Laguna Mucubaji (Venezuela); *** *P. columella *cox1a from Puerto Rico = *L. ubaquensis cox*1a

In a multiple 672-bp-long sequence alignment including the five aforementioned sequences, a total of 565 positions were conserved, 107 variable, 18 parsimony informative and 89 singleton sites. The sequence of *L. cousini *from Chanchu-Yacu (Ecuador) and the two of *L. bogotensis *from Bogota savannah (Colombia) differ in only 3 mutations. The number of nucleotide differences increases considerably to 37 (5.5% divergence) when these three sequences are compared to that of *L. cousini *from Laguna Mucubaji (Venezuela) and to very numerous when with the one shared by *L. ubaquensis *and *P. columella *(Figure 5).

**Figure 6:**
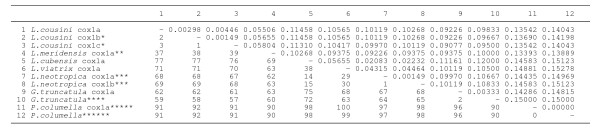
**Pairwise distances between mtDNA *cox*1 nucleotide sequences according to PAUP. Including the lymnaeid species studied, together with Latinoamerican species of the *Galba*/*Fossaria *group and other proximal lymnaeid species available in GenBank (only *cox*1 sequence fragments of a length similar to that of sequences obtained in present study).** Below diagonal = total character differences; above diagonal = mean character differences (adjusted for missing data). Haplotype codes only provisional due to incomplete sequences of the gene. Sequence correspondences: * *L. cousini cox*1b and *cox*1c correspond to two different haplotypes found in *L. bogotensis*; ** *L. meridensis *n. sp. = *L. cousini *from Laguna Mucubaji (Venezuela); *** *L. neotropica *cox1a and cox1b from Peru and Argentina, respectively; **** *G. truncatula *from Germany, without provisional code ascription due to undetermined nucleotides in the sequence [GenBank: EU818799] [70]; ***** *P. columella cox*1a = *L. ubaquensis*; ****** *P. columella *from Australia, without provisional code ascription due to the shorter sequence fragment [GenBank: AY227366] [54]

Species relationships were analysed by comparing these five *cox*1 sequences with other proximal lymnaeid species available in GenBank, whose *cox*1 fragment sequences were similar in lenght to those obtained in the present paper, in a pairwise *cox*1 distance matrix (Figure 6).

**Figure 7:**
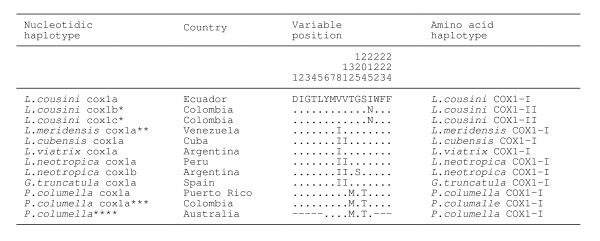
**COX1 amino acid sequence differences detected in the alignment of the haplotypes of the lymnaeid species studied, together with Latinoamerican species of the *Galba*/*Fossaria *group and other proximal lymnaeid species available in GenBank (only *cox*1 sequence fragments of a lenght similar to that of sequences obtained in present study)**. Variable positions = Numbers (to be read in vertical) refer to positions obtained in the alignment made with MEGA 4.0. - = position not sequenced. Haplotype codes only provisional due to incomplete sequences of the gene. Sequence correspondences: * *L. cousini cox*1b and *cox*1c correspond to two different haplotypes found in *L. bogotensis*; ** *L. meridensis *n. sp. = *L. cousini *from Laguna Mucubaji (Venezuela); *** *P. columella cox*1a = *L. ubaquensis*; **** *P. columella *sequence fragment shorter [GenBank: AY227366] [54]

For each of the lymnaeid taxa studied, the amino-acid sequence of the COX1 gene fragment obtained was 224 aa long. A pairwise comparison of the COX1 amino-acid sequences showed a 100% identity between the two *L. bogotensis *from Bogota savannah (Colombia), and only one amino-acid difference (asparagine/isoleucine, respectively) in position 215 between them and *L. cousini *from Chanchu-Yacu (Ecuador). When comparing *L. cousini *from Chanchu-Yacu (Ecuador) and *L. bogotensis *from Bogota savannah (Colombia) with *L. cousini *from Laguna Mucubaji (Venezuela), two differences appear: valine/isoleucine respectively in position 8, and isoleucine in lymnaeids from both Ecuador and Venezuela and asparagine in those from Colombia in position 215. The COX1 amino-acid sequence of *P. columella *is characterised by the presence of a methionine and threonine in positions 32 and 204, whereas all others show threonine and serine, respectively. Worth mentioning is that *L. cousini *from Laguna Mucubaji (Venezuela) shows a COX1 amino-acid sequence identical to that of *L. viatrix *from Argentina (AM494010) (Figure 7).

#### Phylogenetic Analysis

**Figure 8:**
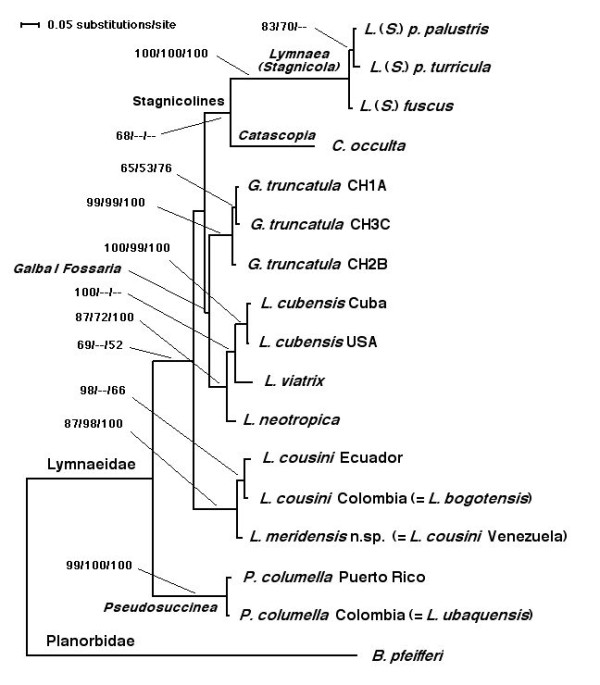
**Phylogenetic maximum-likelihood analysis of lymnaeid species from northern Andean countries**. Phylogenetic tree of lymnaeid species studied, obtained using the planorbid *B. pfeifferi *as outgroup, based on maximum-likelihood (ML) estimates. Scale bar indicates the number of substitutions per sequence position. Support for nodes a/b/c: a: bootstrap with NJ reconstruction using PAUP with ML distance and 1000 replicates; b: bootstrap with ML reconstruction using PAUP with 1000 fast-heuristic replicates; c: Bayesian posterior probability with ML model using MrBayes.

The combination of the two internal transcribed spacers in a single data-set generated a robust tree, indicating phylogenetic accordance between the two spacers. The ML model best fitting this data-set was HKY85+G, using a ts/tv ratio of 1.062 (kappa = 2.08626), base frequencies for A, C, G and T of 0.2094, 0.2696, 0.2405 and 0.2806, respectively, a proportion of invariable sites = 0, and a gamma-distribution shape parameter of 0.53. To assess the reliability of the nodes in the ML tree (Figure 8), a bootstrap analysis using 1000 replicates was made using fast step-wise addition and the neighbor-joining (NJ) algorithm with the ML pairwise distances in PAUP. Finally, a Bayesian phylogeny reconstruction procedure was applied to obtain posterior probabilities (BPP) for the nodes in the ML tree with MrBayes.

In the ML tree obtained (Figure 8), *L. cousini *from Chanchu-Yacu (Ecuador) and *L. bogotensis *from Bogota savannah (Colombia) cluster together with *L. cousini *from Laguna Mucubaji (Venezuela), within a well supported clade (87/98/100 in NJ/ML/BBP). This clade appears basal to the *Galba/Fossaria *species and the European stagnicoline species groups. However, this basal position does not seem to be clearly resolved, given the relatively low supports. *Lymnaea ubaquensis *from Ubaque (Colombia) clusters in the same branch with *P. columella *from Puerto Rico with the highest support, but the relationship of this branch with the main node including all other lymnaeid species is not well resolved. A similar, low-supported link appears between the European *Lymnaea *(*Stagnicola*) species and the basally appearing Palaearctic *C. occulta*, contrary to the relationships between the different *Galba/Fossaria *species which are well supported.

**Figure 9:**
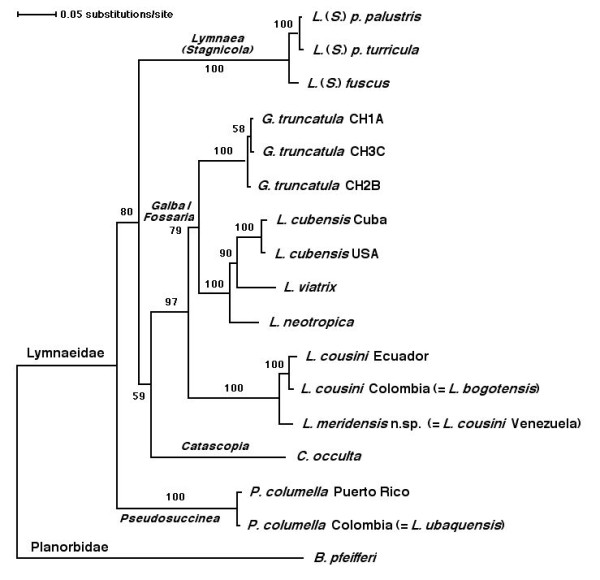
**Phylogenetic neighbor-joining analysis of lymnaeid species from northern Andean countries**. Phylogenetic tree of the lymnaeid species studied, obtained using the planorbid *B. pfeifferi *as outgroup, based on minimum evolution (ME) tree based on Log-Det corrected distances using the neighbor-joining (NJ) method. Scale bar indicates the number of substitutions per sequence position. Numbers indicate the frequency of a particular branch cluster in 1000 bootstrap replicates.

The topology obtained with the NJ algorithm using LogDet distances (Figure 9) is somewhat different to that shown by the ML tree (Figure 8). Here again, *L. cousini *from Ecuador, *L. bogotensis *from Colombia and *L. cousini *from Venezuela cluster together with a 100% bootstrap support, but this branch now appears as a sister group of the *Galba*/*Fossaria *species clade, a 97% of bootstrap value supporting this relationship. As in the ML phylogeny, *L. ubaquensis *from Ubaque (Colombia) clusters with *P. columella *from Puerto Rico in a 100% supported branch which appears basal to the rest of lymnaeids. The position of the Palaearctic *C. occulta *in this topology becomes interestingly different, changing to appear separated from the rest of European stagnicolines and becoming basal to the node including the *Galba*/*Fossaria *species group plus the lymnaeids studied in the present work.

#### Diagnostic Description of *Lymnaea meridensis *n. sp

##### Type locality

A permanent pond in Mucubaji, Merida State, located at an altitude of 3,550 m (8°47'51.8'' N, 70°49'32.4'' W).

##### Other locality

A small ditch in the Paso del Condor area, Merida State, at an altitude of 4,040 m (8°50'38.2.'' N, 70°49'33.9'' W).

##### Type specimens

**Figure 10:**
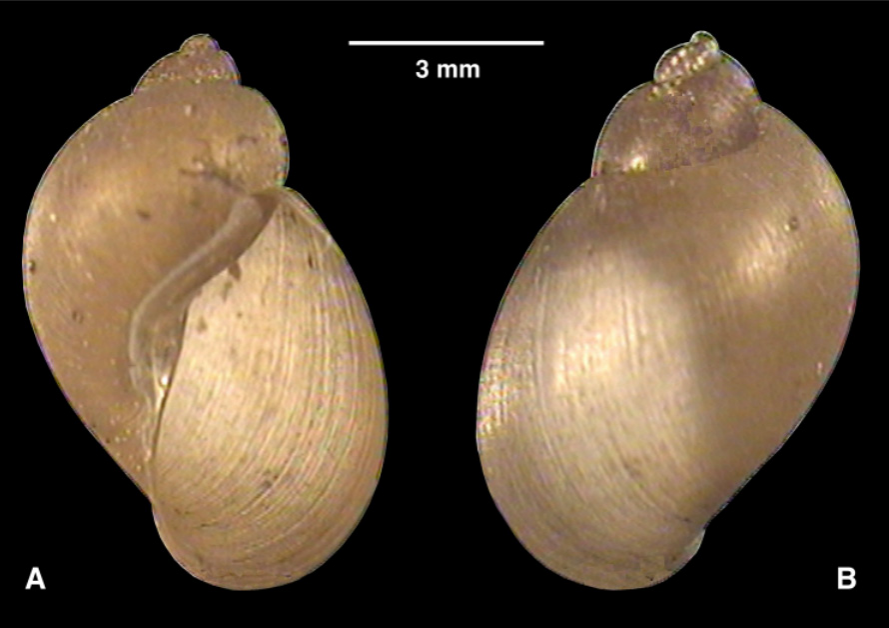
**Shells of *Lymnaea meridensis *n. sp**. Shells of *Lymnaea meridensis *n. sp. from Laguna Mucubaji, Merida State, Venezuela, in ventral (A) and dorsal (B) views.

Voucher specimens are deposited in the parasite and vector collection of the Department of Parasitology, University of Valencia, Valencia, Spain, including a haplotype (Figure 10; 8.5 mm long by 5.4 mm wide; DPUV No. 00.03.20.1.MV) and four paratypes (DPUV No. 00.03.20.2 - 5.MV). In accordance with section 8.6 of the ICZN's International Code of Zoological Nomenclature, copies of this article are deposited at the following five publicly accessible libraries: Natural History Museum, London, UK; American Museum of Natural History, New York, USA; Museum National d'Histoire Naturelle, Paris, France; Russian Academy of Sciences, Moscow, Russia; Academia Sinica, Taipei, Taiwan.

##### Shell

The shell is light brown, thin-walled, with relatively short spire, obtuse apex and fine growth lines, and has only 3 whorls. The body whorl dominates the shell, is inflated and separated by a deep, well-marked suture. The aperture is large, oblique, oval and wide at the base. The form of the shell is illustrated in Figure 10. Measurements and calculated ratios are noted in Table 2. The shell tends to be one and a half times as long as it is wide, and its aperture tends to be two thirds as long as the shell or more than twice as long as the spire.

**Table 2:** Lymnaeid shell measurement comparison between *Lymnaea cousini*, *L. bogotensis *(= *L. cousini*), *L. ubaquensis *(= *Pseudosuccinea columella*) and *L. meridensis *n. sp. (= *L. cousini *from Laguna Mucubaji, Venezuela), from their respective type localities in northern Andean countries

	*L. cousini* Chanchu-Yacu, Chillogallo Ecuador		*L. bogotensis* Savannah of Bogota Colombia		*L. ubaquensis* Laguna de Ubaque Colombia	***L. meridensis *n. sp**. Mucubaji, Mérida State Venezuela
Shell parameters	Jousseaume (1887)^1^ **n = n.s**.	Paraense (1995)^2^ n = 24	Velasquez (2006)^3^ n = 30	present study n = 30	present study n = 30	present study n = 16
Shell length (SL)	10-14	6.6-8.5	3.1-11.7 (6.85 ± 2.3)	4.4-7.2 (6.03 ± 0.65)	7.9-11.8 (9.47 ± 0.97)	6.6-9.3 (8.05 ± 0.78)
Shell width (SW)	5-6/6-10	6.0	7.0	2.9-4.2 (3.64 ± 0.38)	4.6-6.9 (5.49 ± 0.52)	3.7-6.0 (5.24 ± 0.58)
Last spire length (LSL)	n.s.	n.s.	n.s.	3.9-6.3 (5.26 ± 0.56)	7.2-10.8 (8.62 ± 0.89)	6.1-8.4 (7.57 ± 0.13)
Spire length (SpL)	n.s.	3	4.6	1.3-2.5 (1.99 ± 0.26)	1.7-3.2 (2.37 ± 0.30)	1.6-2.7 (2.24 ± 0.31)
Aperture length (AL)	7-10	6	7.1	2.8-4.6 (3.79 ± 0.42)	5.5-8.6 (6.65 ± 0.72)	4.8-6.0 (5.70 ± 0.15)
Aperture width (AW)	4-6	4	5.3	1.9-3.0 (2.53 ± 0.28)	3.4-5.0 (4.06 ± 0.39)	2.5-3.9 (3.39 ± 0.13)
Whorl number	4	5	n.s.	3-4 (3.20 ± 0.41)	3-4 (3.13 ± 0.35)	3-3 (3.00 ± 0.00)
SW/SL ratio	n.s.	0.54-0.65 (0.59 ± 0.03)	0.54-0.71 (0.62 ± 0.05)	0.57-0.67 (0.60 ± 0.02)	0.53-0.65 (0.58 ± 0.03)	0.58-0.66 (0.62 ± 0.04)
AL/SL ratio	n.s.	0.61-0.69 (0.65 ± 0.02)	0.55-0.76 (0.64 ± 0.05)	0.60-0.67 (0.63 ± 0.02)	0.66-0.75 (0.70 ± 0.02)	0.65-0.73 (0.69 ± 0.04)
AL/SpL ratio	n.s.	1.59-2.23 (1.88 ± 0.18)	1.23-3.17 (1.84 ± 0.41)	1.62-2.19 (1.91 ± 0.15)	2.37-3.75 (2.82 ± 0.30)	2.17-3.03 (2.58 ± 0.43)
SpL/SL ratio	n.s.	0.31-0.38 (0.35 ± 0.02)	0.24-0.45 (0.36 ± 0.05)	0.30-0.37 (0.33 ± 0.02)	0.20-0.28 (0.25 ± 0.02)	0.24-0.30 (0.27 ± 0.03)

Range include minimum and maximum extremes, with mean and standard deviation SD in parentheses. Measurements in mm. n.s. = not specified. ^1 ^= [55]; ^2 ^= [40]; ^3 ^= [43]

##### Anatomy

**Figure 11:**
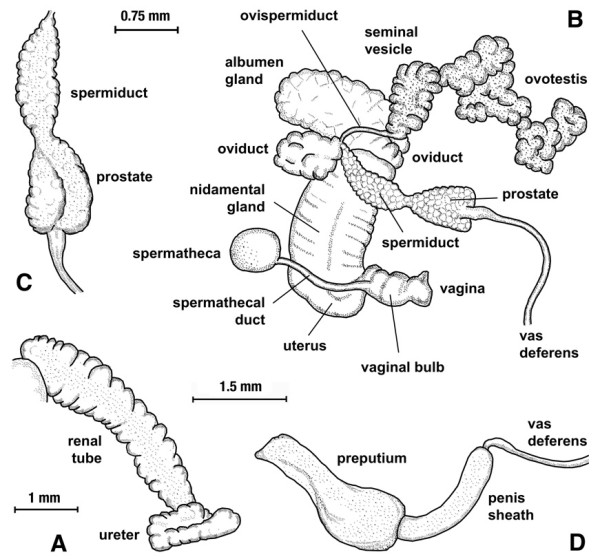
**Anatomy of *Lymnaea meridensis *n. sp**. Aspects of soft part anatomy of *Lymnaea meridensis *n. sp. from Laguna Mucubaji, Venezuela: A) renal tube and ureter in renal region extending between pericardium and mantle collar; B) reproductive system; C) detail of spermiduct and prostate in dorsal view; D) preputium and penis sheath. Scale bars: A = 1 mm; B,D = 1.5 mm; C = 0.75 mm.

The morphoanatomical features are shown in Figure 11. The renal tube extends straightly from the pericardial region toward the mantle collar, diagonally across the roof of the pallial cavity. In its distal part, behind the osphradium, it shows two distinct flexures, coming back upon itself and, after a short course, bending sharply cephalad and rightward forming a ureter which tapers to a subterminal meatus behind the pneumostome (Figure 11A).

The ovotestis appears composed by pressed acini around a collecting canal which continues into an ovispermiduct presenting a very short smooth-walled proximal segment followed by a bosselated swelling seminal vesicle and finally a relatively short distal segment which ends in the carrefour. The albumen gland covers the carrefour and the origin of a bosselated, transverse tubular oviduct which follows a somewhat convolute course continuing into a striated nidamental gland. The nidamental gland narrows into a smooth-walled uterus, which bends and continues into a short bulbous vagina showing a sphincter-like thickening. The spherical spermatheca gives rise to an uniformly thin spermathecal duct which extends diagonally between the nidamental gland and the prostate until joining the vagina (Figure 11B).

The distal portion of the spermiduct and the proximal portion of the prostate run on the ventral surface of the nidamental gland. The spermiduct, of granular outer surface, emerges from the carrefour, runs distalward and finally narrows to merge into a similarly granular prostate (Figure 11B). The prostate increases in width to its distal end, shows ventrally a lengthwise fissure, formed by the folding of its left margin, and finally two rounded protuberances, from whose convergence the vas deferens arises (Figure 11C). The vas deferens appears as a long, more or less uniformly thin duct which merges into a penis which is included within the penial sheath (Figure 11D).

The penis sheath is regularly cylindrical, with a somewhat thicker proximal part. The penis sheath is a little longer than the prepuce (ratio range of 0.93-1.38; mean 1.18 ± 0.18). The prepuce is thicker, around twice as wide as the penis sheath at the point of insertion of the penial sheath and gradually narrowing to terminate in the male genital pore (Figure 11D).

##### DNA sequence markers

Specific classification can be based on the sequences of rDNA ITS-2 (GenBank Accession No. FN598154; haplotype H1), rDNA ITS-1 (FN598159; haplotype HA) and mtDNA *cox*1 (FN598164; provisional haplotype code Ha). For supraspecific classification, the nucleotide sequence of the 18S rRNA gene (FN598151) can be employed. The amino-acid sequence corresponding to the mtDNA COX1 protein (FN598164; provisional haplotype code HI) does not appear to be helpful for species discrimination.

### Discussion

#### Genetic characterisation

The sequences of such a conserved gene as the 18S rRNA [45,46] indicate that *L. cousini *from Chanchu-Yacu (Ecuador), *L. bogotensis *from Bogota savannah (Colombia) and *L. meridensis *n. sp. from Laguna Mucubaji (Venezuela) belong to the same evolutionary lineage and which is different of the one of *L. ubaquensis *from Laguna Ubaque (Colombia) and *P. columella *from Puerto Rico. The surprisingly high number of nucleotide differences in conserved positions of the 18S between our *P. columella *from Puerto Rico and the same species in Argentina [67] indicate that possibly another unknown lymnaeid species was involved in that study carried out in Argentina or, most probably, the Argentinean sequence was not sufficiently "clean". In one or another case, results showing a real-time, 18S-based PCR strategy to be useful for rapid discrimination among main lymnaeid species from Argentina [67] should be reassessed, even if the target used was the sequence fragment corresponding to the helix E10-1 of the variable area V2 highlighted by Bargues and Mas-Coma [46] and not the entire gene. Moreover, this gene has recently proved to be useless for the discrimination of other lymnaeid species present in this country, as *L. viatrix *and *L. neotropica *which present identical 18S sequence [62].

The analyses of both ITS-2 and ITS-1 offer conclusive results. The identical ITS-2 sequence and scarcely different ITS-1 sequences indicate that the lymnaeid populations from Chanchu-Yacu (Ecuador) and Bogota savannah (Colombia) are indeed different combined haplotypes belonging to the same species. Consequently, the systematic name *L. cousini *shall be retained and *L. bogotensis *is molecularly confirmed to be its synonym. This conclusion agrees with the same synonymy based on shell morphology and soft part anatomy already proposed by Hubendick [39] and later accepted by several authors [37,43,44], but different from the proposal of Malek [86] who included *L. bogotensis *as a synonym of *P. columella*. Thus, the population of the type locality Chanchu-Yacu (Ecuador) corresponds to the combined haplotype *L. cousini *ITS-2 H1 and ITS-1 HA, and that of Bogota savannah (Colombia) to *L. cousini *ITS-2 H1 and ITS-1 HB. The total of 16 nucleotide substitutions in ITS-1 (Table 1), despite the lack of differences in ITS-2, suggests that these two populations begin to follow divergent evolutionary lineages, owing to the evolutionary rate of ITS-1 faster than the one of ITS-2 in lymnaeids [45].

However, ITS-2 and ITS-1 sequences do not support the synonymy of *L. ubaquensis *with *L. cousini *proposed by Hubendick [39] and Malek [86]. In fact, the insufficient description of *L. ubaquensis *by both Piaget [57] and Hubendick [39] does not allow to draw clear conclusions on the true identity of this species, and its quotation by Hubendick [39] in such a southern locality as Valdivia, in Chile, has posed acceptance problems [37]. The sequences of the two spacers demonstrate that the population of Laguna Ubaque (Colombia) is only a haplotype of *P. columella*. Therefore, *L. ubaquensis *becomes a synonym of *P. columella *with the following haplotype correspondences: the population of Puerto Rico corresponds to the combined haplotype *P. columella *ITS-2 H1 and ITS-1 HA, and that of Laguna Ubaque (Colombia) to *P. columella *ITS-2 H2 and ITS-1 HA. The sequence of the intergenic ITS-1, 5.8S, ITS-2 region of *P. columella *from Cuba ((AY186751)) [69] should be verified with regard to the 12 indels (9 in ITS-2 and 3 in ITS-1) appearing in the comparison, previously to a definitive haplotype code ascription. Interestingly, these authors were able to find a correlation between two mutations, one in each one of the spacers, and the susceptibility or resistance of *P. columella *populations to *F. hepatica *miracidial infection. With regard to this aspect, *P. columella *from Puerto Rico presents C (susceptible) and that of Laguna Ubaque shows T (resistant) in position 6 of the ITS-2, and both present A (susceptible, instead of G in the resistant populations) in position 510 of the ITS-1.

Additionally, ITS-2 and ITS-1 sequences from Venezuelan lymnaeids are sufficiently different from those of *L. cousini *(= *L. bogotensis*) and *P. columella *(= *L. ubaquensis*) as to indicate that the population of Laguna Mucubaji (Venezuela) merits species status. Although close to *L. cousini*, the 12.65% divergence at ITS-2 level and 11.29-11.73% divergence at ITS-1 level fit within ITS divergences known between different species and are higher than those known between different populations of the same species in Lymnaeidae [8,14,42,48-51]. Thus, these results agree with the phenotypic differences shown by the shell and soft part anatomy (see below) and support the erection of *L. meridensis *n. sp.

The mtDNA *cox*1 sequence fully supports the results obtained with rDNA ITSs (haplotype codes only provisional due to incomplete sequences of the gene - see Figures 5, 6 and 7). The very few nucleotide differences between *L. cousini *from Chanchu-Yacu (Ecuador) and the two of *L. bogotensis *from Bogota savannah (Colombia) indicate their belonging to the same species. The higher differences of the aforementioned with that from *L. cousini *from Laguna Mucubaji (Venezuela) support species status for the latter. The identical *cox*1 sequence of *L. ubaquensis *from Laguna Ubaque (Colombia) and *P. columella *from Puerto Rico also indicates that these lymnaeids belong in fact to the same species. Finally, the great *cox*1 differences between the lymnaeids from Ecuador, Colombia and Venezuela, on one side, and those of Laguna Ubaque and Puerto Rico, on the other side, indicate that two well separated groups are involved.

Differences in the COX1 amino-acid sequence do not appear to be helpful (haplotype codes only provisional due to incomplete sequences of the gene - see Figure 7). The two amino-acid positions discriminating *L. ubaquensis *from Laguna Ubaque (Colombia) and *P. columella *from Puerto Rico from the rest of lymnaeid species seem to be the only exception. Amino-acids in these two positions agree with the incomplete sequence of *P. columella *from Australia [54] (Figure 7). The lack of amino-acid differences between two species as distant one another as *L. cousini *from Laguna Mucubaji (Venezuela) and *L. viatrix *from Argentina [42] suggests that nucleotide saturation in codon positions occurs in the evolution of the mtDNA *cox*1 coding gene.

#### Intergenic region pseudogene exclusion

The numerous, non-microsatellite/minisatellite-related indels found in both ITS-2 and ITS-1 sequences when comparing the populations of *L. cousini *from Chanchu-Yacu (Ecuador) and *L. bogotensis *from Bogota savannah (Colombia), on one side, with those of *L. cousini *from Laguna Mucubaji (Venezuela), on the other side, offered a situation never detected in lymnaeids before. This posed a question mark on the accuracy of the ITS-2 and ITS-1 sequences obtained, even more when considering that these sequences came from different populations of the *a priori *same species.

Although the so-called intergenic region, including the short-length 5.8S rRNA gene separating the two internal transcribed spacers ITS-1 and ITS-2, has been assumed to evolve in concert, the number of investigations revealing high degrees of intra-individual polymorphism has risen in recent years. Such an intra-individual polymorphism is the consequence of an incomplete concerted or non-concerted evolution caused by i.e. hybridization, disadventageous loci or polyploidy [87,88]. In the studies on eukaryotes in which polymorphic intergenic regions have been identified, polymorphic ITS copies have often shown to contain potential pseudogenes in addition to functional copies [89]. Pseudogenes are DNA sequences that (i) were derived from functional genes but have been rendered nonfunctional by mutations that prevent their proper expression and (ii) evolve at a high rate because they are subject to no functional constraints. The influence of pseudogenes is one of the most important issues recently arisen in the debates on phylogenetic hypotheses [80].

When dealing with the intergenic region of the nuclear rDNA, one way to rule out the possibility of pseudogenes being related to *a priori *unexpected sequences of the ITSs is through the 5.8S rDNA sequence. Putative pseudogenes can be identified by the detection of mutations at highly conserved sites such as within the 5.8S [90]. Hence, this gene has become the most valuable indicator of the functionality of ITS copies [91]. The 5.8S is assumed to be highly conserved because its secondary structure is required for proper function of the ribosomal complex [92]. Thus, 5.8S rDNA copies which have lost the ability to build up this conserved secondary structure represent pseudogenes. They are expected to mutate freely, even in conserved positions. Therefore, pseudogenes are characterised by accelerated substitution rates, length variation, methylation-related substitutions causing reduced GC content, and reduced stability of the secondary structure [93-96].

In the present study, sequencing results obtained from the 5.8S rRNA gene of the three aforementioned lymnaeid populations yielded three 154-bp-long sequences which proved to be identical one another. The 5.8S rDNA proved to be a conserved sequence, with no difference in length, total absence of mutations and no low GC content. All these features corroborate that no pseudogenes are involved in the numerous indels appearing between the ITS-2 and ITS-1 sequences. Hence, there is no reason against the presence of that high non-microsatellite/minisatellite-related indel polymorphism in functional ITS sequences of the three lymnaeid populations in question.

#### Phenotypic differentiation

*Lymnaea cousini *(= *L. bogotensis*; = *L. selli*), *L. meridensis *n. sp. (= *L. cousini sensu *Pointier *et al*., 2004, 2009) and *P. columella *(= *L. ubaquensis*) present a similar general type of shell, with an aperture of about 2/3 of the total shell height, which explains the repeated confusion in specimen classifications. However, a detailed morphometric comparison allows the differentiation of *L. cousini *and *L. meridensis *n. sp. by three shell characteristics (Table 2; Figure 10): (i) the shell in *L. cousini *is of greater size, of up to 14/10 mm, 13.7/8.6 mm and 11.7/7.0 mm length/width according to different studies [43,55,56], than in *L. meridensis *n. sp. (up to 9.3/6.0 mm length/width), (ii) it has 4 whorls in *L. cousini *at a shell length in which there are only 3 whorls in *L. meridensis *n. sp., and (iii) the spire is relatively shorter in *L. meridensis *n. sp. than in *L. cousini *(which is shown by a greater AL/SpL ratio in the new species). In its turn, both *L. cousini *and *L. meridensis *n. sp. differ from *P. columella *by the characteristic raised spiral threads of the periostracum, which are absent in the former two lymnaeid species but present in *P. columella *[86]. Additionally, the shell of *L. cousini *and *L. meridensis *n. sp. is more broadly conic, its aperture wider and the body whorl more convex than in *P. columella*.

With regard to the inner anatomy, four characteristics may help in distinguishing *L. meridensis *n. sp. from *L. cousini*: a) the distal segment of the ovispermiduct or hermaphroditic duct appears to be shorter in *L. meridensis *n. sp. than in *L. cousini *[40,43]; b) the spermiduct is only somewhat thinner than the prostate in *L. meridensis *n. sp. whereas clearly slender in *L. cousini *[40,43]; c) the external forms at the beginning of the penis sheath, described as a circlet of minute knobs by Paraense [40] and as a ring of papillae by Velasquez [43], were not observed in *L. meridensis *n. sp.; d) the penis sheath length/prepuce length ratio in *L. meridensis *n. sp. (range of 0.93-1.38; mean 1.18 ± 0.18) is smaller than in *L. cousini *(penis sheath is 1.5 times as long as the prepuce according to Paraense [40]; range of 1.30-1.96; mean 1.5 ± 0.24 according to Velasquez [43]).

Moreover, *L. cousini *shares several crucial anatomic features with *L. meriden*sis n. sp. but which allow them to be distinguished from *P. columella *and other important authochthonous lymnaeid vector species in South America [40,42,97-100): A) Kidney: it distally presents two distinct flexures in the ureter, similarly as in *P. columella *but differently than in *L. viatrix*, *L. neotropica *and *L. diaphana *in which this distal part is straight; B) Vagina: with a bulbous swelling, which is absent in *P. columella*, *L. viatrix*, *L. neotropica *and *L. diaphana*; C) Spermiduct: thinner than the prostate, as in *L. viatrix*, *L. neotropica *and *L. diaphana*, whereas of about the same width in *P. columella*; D) Prostate: with distal oblique or lengthwise fissure, as in *L. viatrix*, *L. neotropica *and *L. diaphana*, whereas such a fissure is absent in *P. columella*; E) Penis sheath: from as long to longer than the prepuce, while much shorter in *P. columella*, shorter in *L. viatrix *and *L. neotropica*, and from as long to shorter in *L. diaphana*.

#### Species relationships

DNA sequences, phenotypic characteristics and the phylogenetic reconstruction show the close relationships between *Lymnaea cousini *(= *L. bogotensis*; = *L. selli*) and *L. meridensis *n. sp., as well as their distance regarding *P. columella *(= *L. ubaquensis*). This means that the assignment of both *L. cousini *and *L. meridensis *n. sp. to the genus *Pseudosuccinea *does not appear to be the correct option, despite their pronounced external resemblance which suggests an evolutionary phenotypic convergence probably related to the inhabitance of similar environments and which has given rise to frequent specimen misclassification as shown in the present study.

Moreover, the phylogenetic relationships between the different great lineages, including the groups of the stagnicolines, the *Galba*/*Fossaria *and *Pseudosuccinea*, and the *L. cousini*-*L. meridensis *group do not appear to be well resolved (see Figures 8 and 9). Consequently, prudence suggests to better keep *L. cousini *and *L. meridensis *n. sp. within the genus *Lymnaea sensu lato *for the time being, awaiting a general review of Lymnaeidae from Latin America which will include the appropriate systematic-taxonomic analysis of the taxa which have been recognised as valid after accurate DNA sequence study. At any rate, according to the value of the information furnished by 18S rDNA sequences [45,46], the results here obtained suggest that the *L. cousini*-*L. meridensis *group is following a lineage different from those of the other lymnaeids hightherto analysed. The ML and NJ-LogDet phylogenetic reconstructions obtained indicate in the same sense.

The present results show that ITS-2, ITS-1 and *cox*1 are good markers not only for identifying *L. cousini*, *L. meridensis *n. sp. and *P. columella *in fascioliasis endemic areas in northern Andean countries, but also for the classification of samples of these species to haplotype level. This usefulness becomes crucial when considering that these three species may be easily confused and specimen classification be a hard task. Numerous exhaustive studies on single nucleotide polymorphisms (SNP) have already proved the value of these three markers for the distinction and identification of lymnaeids. Moreover, they can be helpful in assessing the fascioliasis vector role of different populations, according to recent studies having shown different susceptibility to *F. hepatica *infection, as proved in *P. columella *[69].

#### Distributional outline

Although *L. cousini *appears to be widespread where present [25], it has been the objective of studies published in the literature only sporadically. The southern-most report of this species, under the name of *L. ubaquensis*, in Valdivia, Chile [39] poses a problem already highlighted [37,40]. The brief description of the shell and the male copulatory organ made by Hubendick [39] is insufficient to draw clear conclusions on the correct classification of the Chilean specimen. Although the climate of the lowland of Valdivia is of temperate type and thus not so different from the environmental characteristics of the high altitude in the Andean areas of Ecuador and Colombia, such a southern isolated location becomes surprising and should be verified. The confusion of *L. ubaquensis *with *P. columella *demonstrated in our molecular study suggests that another species may be involved.

Thus, the aforementioned report excluded, the following southernmost report of *L. cousini *concerns Ecuador, namely quoted as *L. raphaelis *Jousseaume, 1887, in the area of Azuay, South of Cuenca (2550 m a.s.l.) [55] and which has also been synonymized with *L. cousini *[39]. Although this finding still needs appropriate confirmation, the distance from and Andean plateau characteristics similar to the more northern Machachi, Pichincha province, Ecuador (78°30'W, 00°30'S, 3100 m a.s.l.) [24] suggests, together with the type locality about 10 km southwest of Quito (2650 m a.s.l.) [40,41,55], that *L. cousini *may be widespread from the south to the north throughout the Andean flatlands in that country. This species has been noted to be also found in Lake San Pablo (0°13' N, 78°14' W, 2660 m a.s.l.), close to Otavalo [41], where it coexists with *P. columella *[101] with which it may be easily confused when only basing on shell characteristics.

In Colombia, Laguna de Ubaque [57] excluded due to the proved confusion of *L. ubaquensis *with *P. columella*, *L. cousini *has been reported, under the name *L. bogotensis*, from the neighbourhood of Bogota city (4°35'56'' N, 74°04'51'' W; 2650 m a.s.l.) [25,27,39,44,56,86] up to the 90 km north from the capital, in the surroundings of Utabe, and also 180 km more northward in Quebrada and Vereda la Toibita in Paipa, Boyaca (5°47'04'' N, 73°06'47'' W; 2525 m a.s.l.) [36,43].

**Figure 12:**
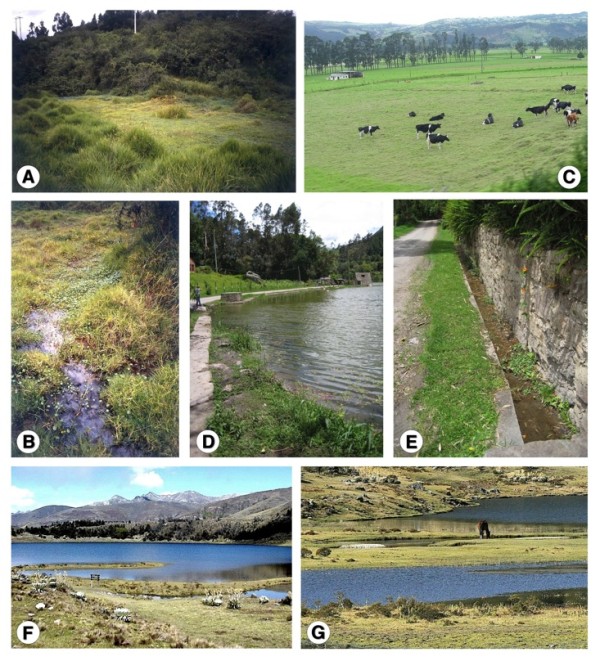
**Lymnaeid biotopes**. Environments of localities where lymnaeids were collected: A, B) habitat of *Lymnaea cousini *in Chanchu-Yacu, Chillogallo, Quito, Ecuador; C) habitat of *L. cousini *(= *L. bogotensis*) in Bogota savannah, Cundinamarca, Colombia; D, E) habitats of *Pseudosuccinea columella *(= *L. ubaquensis*) in Laguna de Ubaque, Cundinamarca, Colombia; F, G) habitat of *L. meridensis *n. sp. in Laguna Mucubaji, Merida State, Venezuela.

Thus, the distribution of *L. cousini *appears restricted to Andean highland areas of an altitude between 2,500 and 3,100 m (Figure 1). This lymnaeid species appears to prefer wet flatlands, where it is typically found among watercress and other aquatic and semiaquatic vegetation in slowly flowing waters of swamped areas from subsoil effluences, as in Chanchu-Yacu [40] (Figure 12A, B), on mud around small watercourses, on the very small water bodies originated in cattle footprints, and on natural and man-made, little deep drainage canals in culture fields and livestock pasturelands in both the savannah of Bogota and Utabe surroundings ([25] and personal observations) (Figure 12C), and more rarely at the water's edges of lakes, lagoons or ponds, as in Lake San Pablo [41].

*Lymnaea meridensis *n. sp. appears, on the contrary, to be a geographically more restricted species according to present knowledge which suggests this species to have evolved isolatedly in permanent ponds and small ditches in more northern, very high altitude areas (3,550-4,040 m) of the Andean mountains, as those of Merida State, in Venezuela [38,41] (Figure 12F, G).

#### Involvement in fascioliasis transmission

*Lymnaea cousini *is known to be a vector of *F. hepatica *since early last century, when it was proved to be the lymnaeid responsible for the usual livestock infection in the Cundinamarca-Boyaca plateau, where it follows an apparent seasonal population dynamics which still need an accurate follow-up analysis. In that area, natural liver fluke infection rates in the snails seem to be low, of 0.07-1.64% [25-27]. This lymnaeid was noted to be a mollusc of low parasitic efficacy for *F. hepatica*, due to its very low fasciolid infection percentages and its additional role of intermediate host for other trematode species [25]. The very low experimental infection rate of 0.6% obtained in *L. cousini *by Muñoz Rivas [27] supported that assumption. However, in an area of the same Bogota savannah presenting a serious animal infection problem, a higher 12% natural infection rate in lymnaeids was found [102]. Although these snails were not classified at specific level, they all presumably belonged to *L. cousini *given their geographical origin. Specimens of the same lymnaeid species were also found infected in Paipa [43].

A surprisingly high natural infection rate of 31.43% was found in *L. cousini *from Machachi, in Ecuador [24], the classification of the fluke larval stages being confirmed by experimental infection of mice with metacercariae [103]. The environmental conditions favouring the development of snail populations and the transmission of the parasite in that Andean area were highlighted to explain such a high transmission rate. The relatively high infection rates of 34.0% and of 39-60% recently obtained in the laboratory with a Colombian isolate of the same lymnaeid species from Vereda la Toibita in Paipa, Boyaca [36], indicate that *L. cousini *may develop an important transmission role in concrete areas.

A recent comparison of experimental infection in *L. cousini *and *P. columella *has furnished interesting results [36]: A) Infectivity: the infection rate in *L. cousini *(34.0%) is pronuncedly lower than that obtained in *P. columella *(82.8% in that study; even higher rates have been obtained in other places [104]). B) Survival: in *L. cousini*, a statistically significant, pronounced decline from 13 weeks post-exposure onwards was detected, whereas numbers of the controls fell drastically only after 25 weeks post-exposure. The mean lifespan of *L. cousini *is long (mean 20-23 weeks) and allows the parasite to remain and produce a great number of rediae. On the contrary, the infection did not affect the survival of *P. columella*, whose lifespan is shorter (mean 9-11 weeks) with regard to the occurrence of cercarial shedding (7 weeks post-exposure). C) Fecundity: parasitisation showed a greater effect on *L. cousini *than on *P. columella*. In *L. cousini*, egg number and mainly egg clusters per snail decreased, egg cluster production ceasing almost completely in infected snails. Influences were also seen in *P. columella*, including a significant reduction in the number of eggs per cluster, mainly egg clusters per snail, and also eggs per snail, many clusters lacking eggs, a phenomenon not observed in *L. cousini*. D) Growth: whereas no effect was observed in *L. cousini*, a reduced growth rate was shown by *P. columella *throughout snail's life. E) Life table: differences appeared smaller in *L. cousini *than in *P. columella*, with a net reproductive rate (*R*o) in the control group of almost double in the first species and nearly four times greater in the second. Thus, the main conclusion is that, although both *L. cousini *and *P. columella *are affected by *F. hepatica *infection, in *L. cousini *the prevalences are low but cercarial shedding is more prolonged since its lifespan is greater, while in *P. columella *infection rates are high but cercarial shedding time is brief due to its short lifespan [36].

The aforementioned compensatory development strategy followed by the liver fluke in *L. cousini *may explain how it is able to maintain high livestock prevalences by its own, as is the case of the 90% prevalence in cattle of Machachi, Ecuador [103] and the high prevalences known in cattle in the Cundinamarca-Boyaca plateau, Colombia [25]. However, this lymnaeid species does not appear to be a vector facilitating human infection, contrary to the case of the species of the *Galba*/*Fossaria *group [4,42]. Indeed, human infection cases reported in areas inhabited by *L. cousini *in both Ecuador [22,23] and Colombia [26,28,29,31] only seem to concern sporadic patients.

With regard to *L. meridensis *n. sp., its phylogenetic relationships and body size suggest that it may most probably be susceptible for *F. hepatica *infection. Its isolated populations on very high altitude areas of Andean Venezuela (3,550-4,040 m) may be involved in disease transmission in such altitude areas during the yearly window in which temperatures are higher than the *F. hepatica *development threshold of 10°C [11]. This is the case of the human hyperendemic area of the Northern Bolivian Altiplano, at 3800-4100 m a.s.l., where disease transmission is increased due to the very high altitude conditions [14] and the highest human prevalences and intensities are known [15,105]. An experimental *F. hepatica *infection assay in the laboratory is needed, and field studies should be carried out to assess its possibly wider distributional outline and potential role to participate in fascioliasis transmission to both animals and humans.

### Conclusion

DNA sequences confirm the originality of the lymnaeid fauna in the northern Andean countries of Ecuador, Colombia and Venezuela. However, results obtained change the species spectrum previously known. *Lymnaea cousini *proved to be a valid species, but *L. bogotensis *and *L. ubaquensis *showed to in fact only be synonyms of *L. cousini *and *P. columella*, respectively. Additionally, a new species apparently endemic to very high altitudes, *L. meridensis *n. sp., should be included in this northern fauna.

The importance of the two original faunistic members lies on their capacity to participate in fascioliasis transmission. *Lymnaea cousini *is involved as vector in highlands of Ecuador and Colombia, where it appears mainly related to animal fascioliasis and only sporadically with isolated human infection cases. Phylogenetic results indicating a close relationship between *L. cousini *and *L. meridensis *n. sp. and their relationship with the *Galba*/*Fossaria *vector group clade, suggest the new species to also be a disease transmitter.

## Competing interests

The authors declare that they have no competing interests.

## Authors' contributions

MDB contributed to the design of the study, participated in field collections, analysed the sequences, performed the phylogenetic study, and helped to draft the manuscript. PA carried out the DNA sequencing processes. MK performed the phenotypic studies on lymnaeid vectors. SMC designed and supervised the study, participated in field collections, performed the epidemiological analyses, and wrote the manuscript. All authors read and approved the final manuscript.

## References

[B1] Mas-Coma S Valero MA Bargues MD *Fasciola*, lymnaeids and human fascioliasis, with a global overview on disease transmission, epidemiology, evolutionary genetics, molecular epidemiology and control Adv Parasitol 2009 69 41 146 19622408 10.1016/S0065-308X(09)69002-3

[B2] Torgerson P Claxton J Dalton JP Epidemiology and control Fasciolosis 1999 Wallingford, Oxon, UK: CAB International Publishing 113 149

[B3] World Health Organization Control of foodborne trematode infections WHO Techn Rep Ser 1995 849 1 157 7740791

[B4] Mas-Coma S Epidemiology of fascioliasis in human endemic areas J Helminthol 2005 79 207 216 10.1079/JOH2005296 16153314

[B5] Mas-Coma S Bargues MD Valero MA Fascioliasis and other plant-borne trematode zoonoses Int J Parasitol 2005 35 1255 1278 10.1016/j.ijpara.2005.07.010 16150452

[B6] Valero MA Mas-Coma S Comparative infectivity of *Fasciola hepatica *metacercariae from isolates of the main and secondary reservoir animal host species in the Bolivian Altiplano high human endemic region Folia Parasitol 2000 47 17 22 10833011 10.14411/fp.2000.004

[B7] Valero MA Darce NA Panova M Mas-Coma S Relationships between host species and morphometric patterns in *Fasciola hepatica *adults and eggs from the Northern Bolivian Altiplano hyperendemic region Vet Parasitol 2001 102 85 100 10.1016/S0304-4017(01)00499-X 11705655

[B8] Bargues MD Vigo M Horak P Dvorak J Patzner RA Pointier JP Jackiewicz M Meier-Brook C Mas-Coma S European Lymnaeidae (Mollusca: Gastropoda), intermediate hosts of trematodiases, based on nuclear ribosomal DNA ITS-2 sequences Inf Genet Evol 2001 1 85 107 10.1016/S1567-1348(01)00019-3 12798024

[B9] Ollerenshaw CB Smith LP Meteorological factors and forecasts of helminthic disease Adv Parasitol 1969 7 283 232 4935270 10.1016/s0065-308x(08)60437-6

[B10] Ollerenshaw CB Taylor AR, Muller R Forecasting liver-fluke disease The Effects of Meteorological Factors upon Parasites 1974 12 Oxford: Symposium of the British Society for Parasitology, Blackwell Scientific Publications 33 52

[B11] Fuentes MV Valero MA Bargues MD Esteban JG Angles R Mas-Coma S Analysis of climatic data and forecast indices for human fascioliasis at very high altitude Ann Trop Med Parasitol 1999 93 835 850 10.1080/00034989957844 10715678

[B12] Mas-Coma S Valero MA Bargues MD Effects of climate change on animal and zoonotic helminthiases Rev Sci Techn Off Int Epiz 2008 27 443 457 18819671

[B13] Mas-Coma S Valero MA Bargues MD Climate change effects on trematodiases, with emphasis on zoonotic fascioliasis and schistosomiasis Vet Parasitol 2009 163 264 280 10.1016/j.vetpar.2009.03.024 19375233

[B14] Mas-Coma S Funatsu IR Bargues MD *Fasciola hepatica *and lymnaeid snails occurring at very high altitude in South America Parasitology 2001 123 S115 S127 11769277 10.1017/s0031182001008034

[B15] Mas-Coma S Angles R Esteban JG Buchon P Franken M Strauss W The Northern Bolivian Altiplano: a region highly endemic for human fascioliasis Trop Med Int Health 1999 4 454 467 10.1046/j.1365-3156.1999.00418.x 10444322

[B16] Esteban JG Flores A Angles R Strauss W Aguirre C Mas-Coma S A population-based coprological study of human fascioliasis in a hyperendemic area of the Bolivian Altiplano Trop Med Int Health 1997 2 695 699 10.1046/j.1365-3156.1997.d01-356.x 9270738

[B17] Esteban JG Gonzalez C Bargues MD Angles R Sanchez C Naquira C Mas-Coma S High fascioliasis infection in children linked to a man-made irrigation zone in Peru Trop Med Int Health 2002 7 339 348 10.1046/j.1365-3156.2002.00870.x 11952950

[B18] Apt W Aguilera X Vega F Alcaino H Zulantay I Apt P Gonzalez V Retamal C Rodriguez J Sandoval J Prevalencia de Fascioliasis en humanos, caballos, cerdos y conejos silvestres en tres provincias de Chile Bol Of Sanit Panam 1993 115 405 414 8274227

[B19] Claxton JR Zambrano H Ortiz P Amoros C Delgado E Escurra E Clarkson MJ The epidemiology of fasciolosis in the inter-Andean valley of Cajamarca, Peru Parasitol Int 1997 46 281 288 10.1016/S1383-5769(97)00039-1

[B20] Ortiz P Cabrera M Jave J Claxton J Williams D Human fascioliasis: prevalence and treatment in a rural area of Peru Infect Dis Rev 2000 2 42 46

[B21] Espinoza JR Maco V Marcos L Saez S Neyra V Terashima A Samalvides F Gotuzzo E Chavarry E Huaman C Bargues MD Valero MA Mas-Coma S Evaluation of Fas2-ELISA for the serological detection of *Fasciola hepatica *infection in humans Am J Trop Med Hyg 2007 76 977 982 17488926

[B22] Trueba G Guerrero T Fornasini M Casariego I Zapata S Ontaneda S Vasco L Detection of *Fasciola hepatica *infection in a community located in the Ecuadorian Andes Am J Trop Med Hyg 2000 62 518 11220770 10.4269/ajtmh.2000.62.518

[B23] Gozalbo M Trueba G Fornasini M Fuentes MV Bargues MD Esteban JG Mas-Coma S Mas-Coma S, et al Coproparasitological survey in schoolchildren from the community of Planchaloma (Province of Cotopaxi, Ecuador) Multidisciplinarity for Parasites, Vectors and Parasitic Diseases, EMOP 9 2004 874 Valencia: J Aguilar SL 447

[B24] Villavicencio A Carvalho de Vasconcellos M First report of *Lymnaea cousini *Jousseaume, 1887 naturally infected with *Fasciola hepatica *(Linnaeus, 1758) (Trematoda: Digenea) in Machachi, Ecuador Mem Inst Osw Cruz 2005 100 735 737 10.1590/S0074-02762005000700010 16410961

[B25] Brumpt E Velasquez J Ucroz H Brumpt LCh Mission E. Brumpt et L.-Ch. Brumpt en Colombie et au Venezuela. 1.- Découverte de l'hôte intermédiaire, *Limnaea bogotensis *Pilsbry, de la grande douve, *Fasciola hepatica*, en Colombie Ann Parasitol Hum Comp 1939 17 563 579

[B26] Muñoz-Rivas G Coccidiosis y distomatosis humanas en Colombia Rev Fac Med Bogotá 1952 21 47 58 13004474

[B27] Muñoz-Rivas G Fasciolosis experimental Rev Acad Colomb Cienc Ex Fís Nat 1953 9 156 158

[B28] Campo Posada A De Castro Gomez F Distomatosis humana. Un nuevo caso en Colombia Rev Hosp San Juan de Dios 1955 61 61 63

[B29] Escobar JA Amezquita-Meneses M Primer caso de *Fasciola hepatica *en el Valle de Cauca Acta Médica del Valle 1973 4 57 58

[B30] Griffiths IB Parra DG Vizcaino OG Gallego MI Prevalence of parasite eggs and cysts in faeces from dairy cows in Colombia Trop Anim Health Prod 1986 18 155 157 10.1007/BF02359526 3765115

[B31] Corredor A Ronderos M Fascioliasis humana en la vereda de Sabaneta, municipio de La Vega, Cundinamarca II Congreso Latinoamericano y V Congreso Colombiano de Medicina Tropical,, Resúmenes. Biomédica 1987 Supl. 1 69

[B32] Gomez T Ciclo de vida de *Fasciola hepatica *(Linnaeus, 1758) e identificación de su huésped intermediario en algunas zonas ganaderas del depatamento del Tolima Rev Univ Tolima, Cienc Tecnol 1990 5 45 75

[B33] Morales G Pino LA Infection de *Lymnaea cubensis *par *Fasciola hepatica *dans une région d'altitude, au Venezuela Ann Parasitol Hum Comp 1983 58 27 30 6870096 10.1051/parasite/1983581027

[B34] Alarcon de Noya B Rojas E Colmenares C Morales C Contreras R Valero SK Hernandez D Briceño S Scorza JV Noya O Brote familiar de fascioliasis en Venezuela Bol Malariol Salud Anim 2007 47 47 54

[B35] Puls-Van der Kamp GM Jansen HB Observations on fascioliasis and its intermediate host, *Lymnaea cousini*, in the Andes mountains of Ecuador Tijdschr Diergeneeskunde 1974 99 410 420

[B36] Salazar L Estrada VE Velasquez L Effect of the exposure to *Fasciola hepatica *(Trematoda: Digenea) on life history traits of *Lymnaea cousini *and *Lymnaea columella *(Gastropoda: Lymnaeidae) Exp Parasitol 2006 114 77 83 10.1016/j.exppara.2006.02.013 16564046

[B37] Pointier JP Noya O Amarista M Theron A *Lymnaea cousini *Jousseaume, 1887 (Gastropoda: Lymnaeidae): first record for Venezuela Mem Inst Osw Cruz 2004 99 567 569 10.1590/s0074-02762004000600005 15558164

[B38] Pointier JP Noya O Alarcon de Noya B Theron A Distribution of Lymnaeidae (Mollusca: Pulmonata), intermediate snail hosts of *Fasciola hepatica *in Venezuela Mem Inst Osw Cruz 2009 104 790 796 10.1590/S0074-02762009000500022 19820844

[B39] Hubendick B Recent Lymnaeidae. Their variation, morphology, taxonomy, nomenclature, and distribution Kungliga Svenska Vetenskapsakademiens Handlingar, Fjärde Serien 1951 3 1 223 + 5 pl

[B40] Paraense WL *Lymnaea cousini *Jousseaume, 1887 from Ecuador (Gastropoda: Lymnaeidae) Mem Inst Osw Cruz 1995 90 605 609 10.1590/S0074-02761995000500011

[B41] Paraense WL Planorbidae, Lymnaeidae and Physidae of Ecuador (Mollusca: Basommatophora) Mem Inst Osw Cruz 2004 99 357 362 10.1590/s0074-02762004000400003 15322623

[B42] Bargues MD Artigas P Mera y Sierra RL Pointier JP Mas-Coma S Characterisation of *Lymnaea cubensis*, *L. viatrix *and *L. neotropica *n. sp., the main vectors of *Fasciola hepatica *in Latin America, by analysis of their ribosomal and mitochondrial DNA Ann Trop Med Parasitol 2007 101 621 641 17877881 10.1179/136485907X229077

[B43] Velasquez LE Synonymy between *Lymnaea bogotensis *Pilsbry, 1935 and *Lymnaea cousini *Jousseaume, 1887 (Gastropoda: Lymnaeidae) Mem Inst Osw Cruz 2006 101 795 799 10.1590/s0074-02762006000700015 17160290

[B44] Mas-Coma S *Lymnaea cousini *(Gastropoda: Lymnaeidae) as transmitter of fascioliasis Mem Inst Osw Cruz 2007 102 241 242 10.1590/S0074-02762007005000023 17426894

[B45] Bargues MD Mas-Coma S Reviewing lymnaeid vectors of fascioliasis by ribosomal DNA sequence analyses J Helminthol 2005 79 257 267 10.1079/JOH2005297 16153320

[B46] Bargues MD Mas-Coma S Phylogenetic analysis of lymnaeid snails based on 18S rDNA sequences Mol Biol Evol 1997 14 569 577 9159934 10.1093/oxfordjournals.molbev.a025794

[B47] Bargues MD Mangold AJ Muñoz-Antoli C Pointier JP Mas-Coma S SSU rDNA characterization of lymnaeid snails transmitting human fascioliasis in South and Central America J Parasitol 1997 83 1086 1092 10.2307/3284367 9406784

[B48] Remigio EA Blair D Relationships among problematic North American stagnicoline snails (Pulmonata: Lymnaeidae) reinvestigated using nuclear ribosomal DNA internal transcribed spacer sequences Can J Zool 1997 75 1540 1545 10.1139/z97-779

[B49] Bargues MD Horak P Patzner RA Pointier JP Jackiewicz M Meier-Brook C Mas-Coma S Insights into the relationships of Palaearctic and Nearctic lymnaeids (Mollusca: Gastropoda) by rDNA ITS-2 sequencing and phylogeny of stagnicoline intermediate host species of *Fasciola hepatica* Parasite 2003 10 243 255 14535164 10.1051/parasite/2003103243

[B50] Puslednik L Ponder WF Dowton M Davis AR Examining the phylogeny of the Australasian Lymnaeidae (Heterobranchia: pulmonata: Gastropoda) using mitochondrial, nuclear and morphological markers Mol Phyl Evol 2009 52 643 659 10.1016/j.ympev.2009.03.033 19362157

[B51] Bargues MD Artigas P Jackiewicz M Pointier JP Mas-Coma S Ribosomal DNA ITS-1 sequence analysis of European stagnicoline Lymnaeidae (Gastropoda) Heldia, München 2006 6 29 40

[B52] Remigio EA Blair D Molecular systematics of the freshwater snail family Lymnaeidae (Pulmonata: Basommatophora) utilising mitochondrial ribosomal DNA sequences J Moll Stud 1997 63 173 185 10.1093/mollus/63.2.173

[B53] Remigio EA Molecular phylogenetic relationships in the aquatic snail genus *Lymnaea*, the intermediate host of the causative agent of fascioliasis: insights from broader taxon sampling Parasitol Res 2002 88 687 696 10.1007/s00436-002-0658-8 12107463

[B54] Remigio EA Hebert PD Testing the utility of partial COI sequences for phylogenetic estimates of gastropod relationships Mol Phyl Evol 2003 29 641 647 10.1016/S1055-7903(03)00140-4 14615199

[B55] Jousseaume F Mollusques nouveaux de la République de l'Equateur Bull Soc Zool France 1887 12 165 186

[B56] Pilsbry HA South American land and freshwater mollusks, IX Colombian species Proc Acad Nat Sci Philadelphia 1935 87 83 88

[B57] Piaget J Quelques mollusques de Colombie Mém Soc Neuchâtel Sci Nat 1914 5 265 269

[B58] Harry HW Hubendick B The freshwater pulmonate Mollusca of Puerto Rico Göteborgs Kungliga Svenska Vetenskapsakademiens Handlingar 1964 3 1 93

[B59] Sambrook J Fritsch EF Maniatis T Molecular Cloning. A Laboratory Manual 1989 I, II & III 2 New York: Cold Spring Harbor Laboratory, Cold Spring Harbor 1 1647

[B60] Folmer O Black M Hoch W Lutz R Vrijenhoek R DNA primers for amplification of mitochondrial cytochrome c oxidase subunit I from diverse metazoan invertebrates Mol Mar Biol Biotechnol 1994 3 294 299 7881515

[B61] Sanger F Nicklen S Coulson AR DNA sequencing with chain-terminating inhibitors Proc Natl Acad Sci USA 1977 74 5463 5467 10.1073/pnas.74.12.5463 271968 PMC431765

[B62] Mera y Sierra R Artigas P Cuervo P Deis E Sidoti L Mas-Coma S Bargues MD Fascioliasis transmission by *Lymnaea neotropica *confirmed by nuclear rDNA and mtDNA sequencing in Argentina Vet Parasitol 2009 166 73 79 10.1016/j.vetpar.2009.08.001 19729246

[B63] Thompson JD Higgins DG Gibson TJ CLUSTAL W: improving the sensitivity and progressive multiple sequence alignment through sequence weighting, positions-specific gap penalties and weight matrix choice Nucl Acids Res 1994 22 4673 4680 10.1093/nar/22.22.4673 7984417 PMC308517

[B64] Tamura K Dudley J Nei MB Kumar S MEGA4: Molecular Evolutionary Genetic Analysis (MEGA) sofware version 4.0 Mol Biol Evol 2007 24 1596 1599 10.1093/molbev/msm092 17488738

[B65] Staden R Judge DP Bonfield JK Sequence assembly and finishing methods Meth Biochem Anal 2001 43 302 322 10.1002/0471223921.ch13 11449730

[B66] Swofford DL PAUP*: phylogenetic analysis using parsimony (*and other Methods) Version 4 2002 Computer program distributed by the Smithsonian Institution. Sunderland, Massachusetts: Sinauer Associates, Inc. Publishers

[B67] Duffy T Kleiman F Pietrokovsky S Issia L Schijman AG Wisnivesky-Colli C Real-time PCR strategy for rapid discrimination among main lymnaeid species from Argentina Acta Trop 2009 109 1 4 10.1016/j.actatropica.2008.08.003 18983808

[B68] Vinarski VM Glöer P Taxonomic notes on Euro-Siberian freshwater molluscs. 3. *Galba occulta *Jackiewicz, 1959 is a junior synonym of *Limnaea palustris *var. *terebra *Westerlund, 1885 Mollusca 2008 26 175 185

[B69] Gutierrez A Pointier JP Fraga J Jobet E Modat S Perez RT Yong M Sanchez J Loker ES Theron A *Fasciola hepatica*: identification of molecular markers for resistant and susceptible *Pseudosuccinea columella *snail hosts Exp Parasitol 2003 105 211 218 10.1016/j.exppara.2003.12.006 14990314

[B70] Albrecht C Wolf C Gloer P Wilke T Concurrent evolution of ancient sister lakes and sister species: the freshwater gastropod genus *Radix *in lakes Ohrid and Prespa Hydrobiologia 2008 615 157 167 10.1007/s10750-008-9555-1

[B71] Winnepenickx B Backeljau T Van De Peer Y De Wachter R Structure of the small ribosomal subunit RNA of the pulmonate snail, *Limicolaria kambeul*, and phylogenetic analysis of the Metazoa FEBS Letters 1992 309 123 126 10.1016/0014-5793(92)81078-Z 1505675

[B72] De Rijk P Neefs JM Van de Peer Y De Wachter R Compilation of small ribosomal subunit RNA sequences Nucl Acids Res 1992 20 2075 2089 1375995 10.1093/nar/20.suppl.2075 PMC333984

[B73] Akaike H A new look at the statistical model identification Ieee Transactions on Automatic Control 1974 19 716 723 10.1109/TAC.1974.1100705

[B74] Posada D Buckley TR Model selection and model averaging in phylogenetics: advantages of the AIC and Bayesian approaches over likelihood ratio tests Syst Biol 2004 53 793 808 10.1080/10635150490522304 15545256

[B75] Posada D Crandall KA Modeltest: testing the model of DNA substitution Bioinformatics 1998 14 817 818 10.1093/bioinformatics/14.9.817 9918953

[B76] Saitou N Nei M The neighbor-joining method: a new method for reconstructing phylogenetic trees Mol Biol Evol 1987 4 406 425 3447015 10.1093/oxfordjournals.molbev.a040454

[B77] Ronquist F Huelsenbeck JP MrBayes 3: Bayesian phylogenetic inference under mixed models Bioinformatics 2003 19 1572 1574 10.1093/bioinformatics/btg180 12912839

[B78] Lin CP Danforth BN How do insect nuclear and mitochondrial gene substitution patterns differ? Insights from Bayesian analyses of combined datasets Mol Phyl Evol 2004 30 686 702 10.1016/S1055-7903(03)00241-0 15012948

[B79] Ballard JWO Rand DM The population biology of mitochondrial DNA and its phylogenetic implications Ann Rev Ecol Evol Syst 2005 36 621 642 10.1146/annurev.ecolsys.36.091704.175513

[B80] Mas-Coma S Bargues MD Populations, hybrids and the systematic concepts of species and subspecies in Chagas disease triatomine vectors inferred from nuclear ribosomal and mitochondrial DNA Acta Trop 2009 110 112 136 10.1016/j.actatropica.2008.10.013 19073132

[B81] DeJong RJ Morgan JAT Paraense WL Pointier JP Amarista M Ayeh-Kumi PFK Babiker A Barbosa CS Bremond Ph Canese AP Pereira de Suoza C Dominguez C File S Gutierrez A Incani RN Kawano T Kazibwe F Kpikpi J Lwambo NJS Mimpfoundi R Njiokou F Poda JN Sene M Velasquez LE Yong M Adema CM Hofkin BV Mkoji GM Loker ES Evolutionary relationships and biogeography of *Biomphalaria *(Gastropoda: Planorbidae) with implications regarding its role as host of the human bloodfluke, *Schistosoma mansoni* Mol Biol Evol 2001 18 2225 2239 11719572 10.1093/oxfordjournals.molbev.a003769

[B82] Oviedo JA Bargues MD Mas-Coma S Lymnaeid snails in the human fascioliasis high endemic zone of the Northern Bolivian Altiplano Res Rev Parasitol 1995 55 35 43

[B83] Samadi S Roumegoux A Bargues MD Mas-Coma S Yong M Pointier JP Morphological studies of lymnaeid snails from the human fascioliasis endemic zone of Bolivia J Moll Stud 2000 66 31 44 10.1093/mollus/66.1.31

[B84] Valero MA Panova M Mas-Coma S Phenotypic analysis of adults and eggs of *Fasciola hepatica *by computer image analysis system J Helminthol 2005 79 217 225 10.1079/JOH2005301 16153315

[B85] Pointier JP Cazzaniga NJ Gonzalez-Salas C Gutierrez A Arenas JA Bargues MD Mas-Coma S Anatomical studies of sibling species within Neotropical lymnaeids snail intermediate hosts of fascioliasis Mem Inst Osw Cruz 2006 101 431 435 10.1590/S0074-02762006000400015 16951816

[B86] Maleck EA Snail Hosts of Schistosomiasis and Other Snail-Transmitted Diseases in Tropical America: A Manual 1985 478 Washington DC: PAHO, Scientific Publication 1 325

[B87] Hughes CE Bailey CD Harris SA Divergent and reticulate species relationships in *Leucanea *(Fabaceae) inferred from multiple data sources: insights into polyploid origins and nrDNA polymorphism Am J Botany 2002 89 1057 1073 10.3732/ajb.89.7.1057 21665706

[B88] Wissemann V Sharma AK, Sharma A Hybridization and the evolution of the nrITS spacer region Plant Genome Biodiversity and Evolution, Part A: Phanerogams 2003 I Enfield: Scientific Publications Inc 57 71

[B89] Harpke D Peterson A Extensive 5.8S nrDNA polymorphism in *Mammillaria *(Cactaceae) with special reference to the identification of pseudogenic internal transcribed spacer regions J Plant Res 2008 121 261 270 10.1007/s10265-008-0156-x 18373158

[B90] Jobes DV Thien LB A conserved motif in the 5.8S ribosomal RNA (rRNA) gene is a useful diagnostic marker for plant internal transcribed spacer (ITS) sequences Plant Mol Biol Reprod 1997 15 326 334 10.1023/A:1007462330699

[B91] Hershkovitz MA Zimmer EA Hahn WJ Hollingsworth PM, Bateman RM, Cornall RJ Ribosomal DNA sequences and angiosperm systematics Molecular Systematics and Plant Evolution 1999 London: Taylor & Francis 268 326

[B92] Suh YB Thien LB Zimmer EA Nucleotide sequences of the internal transcribed spacers and 5.8S rRNA gene in *Canella winterana *(Magnoliales; Canellaceae) Nucl Acids Res 1992 20 6101 6102 10.1093/nar/20.22.6101 1461743 PMC334481

[B93] Li W Molecular Evolution 1997 Sunderland: Sinauer Associates 1 487

[B94] Buckler IV ES Ippolito A Holtsford TP The evolution of ribosomal DNA: divergent paralogues and phylogenetic implications Genetics 1997 145 821 832 9055091 10.1093/genetics/145.3.821 PMC1207866

[B95] Kita Y Ito M Nuclear ribosomal ITS sequences and phylogeny in East Asian *Aconitum *subgenus *Aconitum *(Ranunculaceae), with special reference to extensive polymorphism in individual plants Plant Systematics and Evolution 2000 225 1 13 10.1007/BF00985455

[B96] Bailey CD Carr TG Harris SA Hughes CE Characterization of angiosperm nrDNA polymorphism, paralogy, and pseudogenes Mol Phyl Evol 2003 29 435 455 10.1016/j.ympev.2003.08.021 14615185

[B97] Paraense WL *Lymnaea viatrix*: a study of topotypic specimens (Mollusca: Lymnaeidae) Rev Brasil Biol 1976 36 419 428

[B98] Paraense WL *Lymnaea columella *in northern Brazil Mem Inst Osw Cruz 1983 78 477 482 10.1590/S0074-02761983000400011

[B99] Paraense WL *Lymnaea diaphana*: a study of topotypic specimens (Pulmonata: Lymnaeidae) Mem Inst Osw Cruz 1984 79 75 81 10.1590/S0074-02761984000100009

[B100] Paraense WL *Lymnaea columella*: two new Brazilian localities in the states of Amazonas and Bahia Mem Inst Osw Cruz 1986 81 121 123 10.1590/S0074-02761986000100016

[B101] Paraense WL *Lymnaea viatrix *and *Lymnaea columella *in the Neotropical region: a distributional outline Mem Inst Osw Cruz 1982 77 181 188

[B102] Parra Florez AD Mateus Valles JG El huésped intermediario de *Fasciola hepatica *y su control con N-tritylmorholine VII Congreso Panamericano de Medicina Veterinaria y de Zootecnia (Bogota, 23-28 July 1973), Resúmenes 1973 Bogota: Asociación Panamericana de Medicina Veterinaria y de Zootecnia 42 43

[B103] Carvalho de Vasconcellos M Villavicencio A Reply Mem Inst Osw Cruz 2007 102 242 243

[B104] Gutierrez A Yong M Perera G Sanchez J Theron A *Fasciola hepatica *(Trematoda: Digenea): its effect on the life history traits of *Pseudosuccinea columella *(Gastropoda: Lymnaeidae) an uncommon interaction Parasitol Res 2002 88 535 539 10.1007/s00436-002-0625-4 12107475

[B105] Esteban JG Flores A Angles R Mas-Coma S High endemicity of human fascioliasis between Lake Titicaca and La Paz valley, Bolivia Trans Roy Soc Trop Med Hyg 1999 93 151 156 10.1016/S0035-9203(99)90289-4 10450437

